# Effect of Fungicide Treatment on Multi-Mycotoxin Occurrence in French Wheat during a 4-Year Period

**DOI:** 10.3390/toxins15070443

**Published:** 2023-07-04

**Authors:** Alexandra Kleber, Christiane Gruber-Dorninger, Alexander Platzer, Clarisse Payet, Barbara Novak

**Affiliations:** 1 DSM-BIOMIN Research Center, 3430 Tulln, Austria; christiane.gruber-dorninger@dsm.com (C.G.-D.); alexander.platzer@dsm.com (A.P.); barbara.novak@dsm.com (B.N.); 2 Bayer SAS, Crop Science, 69009 Lyon, France

**Keywords:** wheat, mycotoxins, trichothecenes, deoxynivalenol, *Fusarium*, *Alternaria*, fungicides, multi-mycotoxin analysis

## Abstract

Wheat represents one of the most widely consumed cereals worldwide. Cultivated in winter and spring, it is vulnerable to an array of different pathogens, including fungi, which are managed largely through the in-field application of fungicides. During this study, a 4-year field investigation (2018–2021) was performed in France, aiming to assess the efficacy of fungicide treatment to reduce mycotoxin contamination in common and durum wheat. Several different commercially available fungicides were applied via sprayers. Concentrations of mycotoxins and fungal metabolites in wheat were determined using a multi-analyte liquid-chromatography–tandem-mass-spectrometry-based method. The highest contamination levels and strongest effects of fungicides were observed in 2018, followed by 2021. A significant fungicide-mediated reduction was observed for the trichothecenes deoxynivalenol, deoxynivalenol-3-glucoside, nivalenol, and nivalenol-3-glucoside. Furthermore, fungicide treatment also reduced levels of culmorin and its hydroxy metabolites 5- and 15-hydroxy-culmorin, as well as aurofusarin. Interestingly, the *Alternaria* metabolite infectopyron was increased following fungicide treatment. In conclusion, fungicide treatment was effective in reducing mycotoxin levels in wheat. However, as complete prevention of mycotoxin contamination was not achieved, fungicide treatment should always be combined with other pre- and post-harvest mycotoxin mitigation strategies to improve food and feed safety.

## 1. Introduction

The Food and Agricultural Organization of the United Nations (FAO) estimated that rice, maize, and wheat constitute a major source of food for 4 billion people, making up approximately 60% of the world’s food energy intake [1]. It is therefore of particular concern that, according to worldwide surveys, up to 80% of agricultural commodities are contaminated with secondary fungal metabolites, so-called mycotoxins [2,3]. In fact, mycotoxins represent—aside from other threats, such as pesticide residues, heavy metals, and alkaloids—a major global risk factor associated with the consumption of cereals and cereal-derived products [4,5]. Depending on the type and the contamination levels, mycotoxins can induce a variety of adverse health effects in both humans and animals. Although numerous mycotoxins and other fungal metabolites are currently known, mitigation strategies focus on those compounds that present the most concern regarding human and animal health. With regard to wheat, these include mainly *Fusarium* mycotoxins, such as deoxynivalenol (DON), zearalenone (ZEN), nivalenol (NIV), fumonisins (FUM), T-2, and HT-2 toxins [6,7,8,9,10,11,12,13].

In addition to the well-known mycotoxins, for which maximum levels and guidance values have largely been established in the European Union [3], the number of other unregulated less-investigated fungal metabolites, so-called “emerging mycotoxins”, have gained great interest in recent years [14,15,16]. The enormous advances of analytical methods, as well as the development of multi-toxin methods (e.g., simultaneous detection of multiple fungal metabolites via liquid chromatography–tandem mass spectroscopy (LC-MS/MS)), have enabled the discovery of a large number of these fungal metabolites [14,17,18]. Some of the most common emerging mycotoxins include the *Fusarium* metabolites enniatins (ENNs), beauvericin (BEA), moniliformin (MON), aurofusarin (AURO), fusaproliferin (FP), fusaric acid (FA), culmorin (CUL), and butenolide (BUT); the Aspergillus metabolites sterigmatocystin (STE) and emodin (EMO); the Penicillium metabolite mycophenolic acid (MPA); and the *Alternaria* metabolites alternariol (AOH), alternariol monomethyl ether (AME), tentoxin (Te), infectopyron (IP), and tenuazonic acid (TeA) [14,16,19,20].

The global occurrence of both well-known and emerging mycotoxins and their presence in diverse sources of food and feed depends on several factors. On the one hand, mycotoxin contamination levels may fluctuate between crop varieties due to differences in resistance to fungal pathogens. In addition, mycotoxin formation is heavily affected by agricultural and storage practices, as well as weather conditions, such as temperature, humidity, and elevated CO_2_ levels [2,21,22,23,24]. Although studies on global mycotoxin occurrence indicate that DON is the most prevalent mycotoxin in wheat, remarkable differences have been observed regarding the type and prevalence of mycotoxin contamination in different parts of the world, underlining the important role of such regional climatic conditions [9,12,25,26,27]. In fact, multiple studies have uncovered high levels of variation in the worldwide mycotoxin occurrence in wheat and cereals. While the average contamination level is often well below legal limits, the concentration range may be wide, with numerous samples exceeding maximum or recommended levels of mycotoxin contamination [2,9,12,25,26,27].

In addition to fluctuations observed with regard to mycotoxin contamination levels, various studies clearly indicate that cereals and other agricultural commodities are often not contaminated with only a single mycotoxin—in fact, co-contamination with several different mycotoxins from various fungal sources is very common [2,12,15,25,26,27,28,29]. For example, a survey carried out in 2015 revealed that 46% of wheat samples were co-contaminated with ≥2 mycotoxins [30]. Furthermore, a recently conducted large-scale global survey of mycotoxin occurrence and co-occurrence in feed revealed that up to 64% of investigated samples were co-contaminated with at least two mycotoxins [2]. In addition, this study reported that combinations of DON, ZEN, and FUM, as well as FUM and aflatoxin B1, were particularly prevalent [2]. Furthermore, an Italian study showed that while 80% of wheat samples were contaminated with at least one mycotoxin, 27% of samples contained two different mycotoxins, and 38% were contaminated with three or more mycotoxins [31]. As additive or synergistic effects of co-occurring mycotoxins have been already shown [28], the co-contamination of samples may exert additional adverse health risk.

In order to effectively manage food safety and economic issues resulting from mycotoxin contamination, appropriate mitigation strategies must be employed at preharvest, harvest, and postharvest stages. Postharvest techniques to minimize mycotoxin contamination include suitable storage conditions and moisture adjustment [32,33], and the use of feed additives that enable biodegradation [34,35,36] or adsorption [37,38] of mycotoxins. Furthermore, the detection and decontamination or disposal and continuous monitoring of potential contamination during processing presents an important mitigation tool [39]. Nevertheless, despite the indispensable nature of such measures, the degree of post-harvest contamination is a direct result of pre-harvest presence of fungal contamination. Thus, approaches to in-field mycotoxin management to prevent mycotoxin contamination are at least equally important [21,22]. This includes several agro-technical practices such as crop selection [40,41,42,43,44,45,46,47,48,49], crop rotation [50,51,52], tillage [53], and fertilization [54]. Furthermore, careful planning of crop planting to avoid high temperature and drought during kernel development and maturation, as well as scheduling of suitable harvest times depending on the physiological stages of plants [21,55,56,57,58], are important pre-harvest strategies to reduce mycotoxin contamination.

Finally, the use of fungicides presents an important mechanism to undermine fungal contamination [4]. Fungicide treatment has been shown to reduce, for example, wheat *Fusarium* infection and DON contamination [59,60,61]. For example, according to a study carried out in Italy [59], treatments with cyproconazole combined with prochloraz, as well as a mixture of tebuconazole and azoxystrobin, led to significant reductions of the FHB disease severity and DON concentration in wheat. Furthermore, investigations such as those published by Yoshida et al. in 2012 [61] show that not only the use of fungicides themselves, but also the timing of fungicide application, is crucial. In fact, the latter study investigated the effect of timing of fungicide application on FHB and the accumulation of deoxynivalenol and nivalenol. The authors demonstrated that fungicide application timing differentially affected FHB and mycotoxin concentration, indicating that fungicide application beyond 20 days after anthesis reduced mycotoxin concentration in matured grain without reducing FHB severity. Application at anthesis, however, was shown to be crucial for reducing FHB. In addition to the effects of fungicides on the rather well-known mycotoxins, such as DON, it has also been shown that contamination levels of emerging and modified mycotoxins are also significantly reduced by treatment with azole fungicides. In a study by Scarpino et al., 2015 [62], a series of field experiments were carried out to evaluate the effect of the azole fungicide, prothioconazole, on the prevalence of emerging mycotoxins in common winter wheat. The authors showed significant reductions of enniatins, aurofusarin, moniliformin, tentoxin, and equisetin contents, thereby underlining that fungicides usually applied to control FHB and DON content also consistently reduce the main emerging mycotoxins of winter wheat in temperate areas.

Thus, this study investigates the effect of fungicide treatment on the contamination levels of mycotoxins and emerging mycotoxins in common and durum wheat samples collected in France over a period of 4 years. To this end, common wheat and durum wheat were treated with different fungicides via sprayers, and the contamination levels of an array of mycotoxins were compared to those of control fields. To our best knowledge, this is the first study to investigate fungicides with regard to their reduction efficacy against such a large array of mycotoxins and emerging mycotoxins.

## 2. Results

### 2.1. Fungicide Treatments

When looking at the effects and biases of each individual fungicide on mycotoxin contents in wheat with PCA and hierarchical clustering, we found no major patterns or differences between the fungicides (Figures S1–S45). Consequently, the different types of fungicides were not analyzed separately.

### 2.2. DON, NIV, and Their Masked Forms

**Figure 1:**
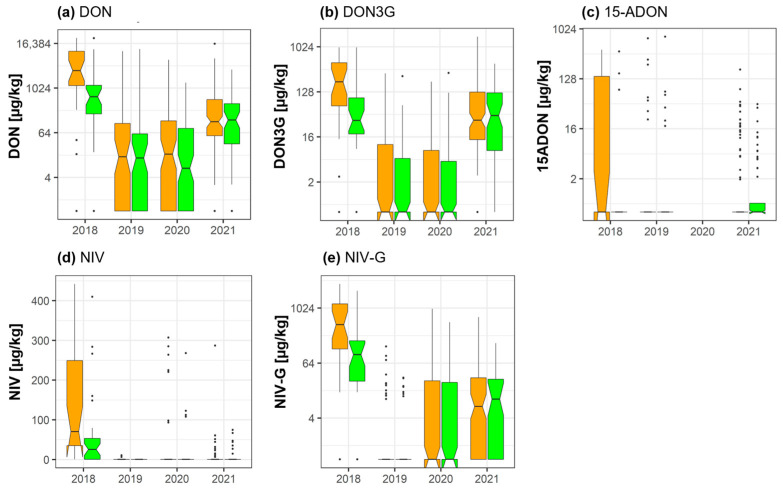
Effect of fungicide treatment on the trichothecenes (**a**) deoxynivalenol (DON), its masked forms (**b**) deoxynivalenol-3-glucoside (DON3G) and (**c**) 15-acetyl deoxynivalenol (15ADON), as well as (**d**) nivalenol (NIV) and its masked form, (**e**) nivalenol-3-glucoside (NIV-3G) during the wheat growing seasons in 2018, 2019, 2020, and 2021 in cereal regions in France. Data represent groupwise analysis. Orange bars indicate control samples; green bars indicate fungicide-treated samples.

Groupwise analysis of the data showed that fungicide treatment led to significant reductions of the mean concentrations of DON (−59.9%) (*p* = 0.044), deoxynivalenol-3-glucoside (DON3G) (−54%) (*p* = 0.044), NIV (−59.2%) (*p* = 0.044), and NIV-3-glucoside (NIV-3G) (−57%) (*p* = 0.044) in 2018. According to this type of data analysis, no statistically significant effects were observed during the remaining years. Concentrations of 3-acetyl-deoxynivalenol (3-ADON) were negligible in all years—the metabolite is therefore not included in Figure 1. Parallel to the groupwise comparison shown in Figure 1, a pairwise analysis of the data (Table S1) indicated significant reductions of DON (*p* = 5.51 × 10^−6^), DON3G (*p* = 0.00031), NIV (*p* = 0.00031), NIV-3G (0.002), and 15-acetyl-deoxynivalenol (15ADON) (0.048) in 2018. Furthermore, according to this type of analysis, fungicide treatment significantly reduced mean concentrations of DON (*p* = 4.13 × 10^−5^), DON3G (*p* = 0.0002), and NIV (*p* = 0.006) in 2020, and DON (*p* = 3.7 × 10^−7^), DON3G (*p* = 1.67 × 10^−7^), 15ADON (*p* = 0.0009), and NIV (*p* = 0.019) in 2021. However, it must be considered that in 2019 and 2020, the overall level of contamination was generally relatively low in both the control and fungicide-treated groups.

### 2.3. Culmorin and Its Derivatives

**Figure 2:**
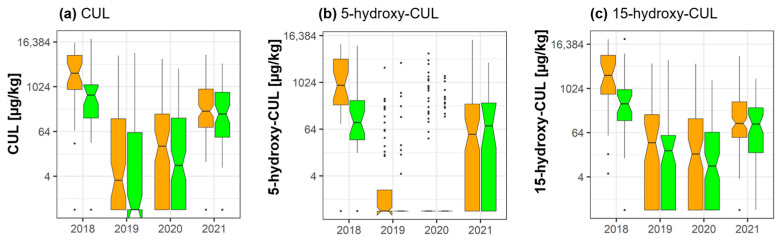
Effect of fungicide treatment on (**a**) culmorin (CUL) and its derivatives (**b**) 5-hydroxy-culmorin (5-hydroxy-CUL) and (**c**) 15-hydroxy-culmorin (15-hydroxy-CUL] during the wheat growing seasons in 2018, 2019, 2020, and 2021 in cereal regions in France. Data represent groupwise analysis. Orange bars indicate control samples; green bars indicate fungicide-treated samples.

Statistically significant fungicide-induced reductions of the mean concentrations of CUL (−54.2%) (groupwise analysis: *p* = 0.046; pairwise analysis: *p* = 0.0005), 5-hydroxy-CUL (−62%) (groupwise analysis: *p* = 0.044; pairwise analysis: 0.0003), and 15-hydroxy-CUL (−57.3%) (groupwise analysis: *p* = 0.044; pairwise analysis: *p* = 0.00003) were detected in 2018 (Figure 2).

In 2020 and 2021, statistically significant fungicide-induced reductions of CUL, 5-hydroxy-CUL, and 15-hydroxy-CUL were only detected in the pairwise data analysis (2020: CUL (*p* = 0.0002), 5-hydroxy-CUL (*p* = 0.0005), 15-hydroxy-CUL (*p* = 4.13 × 10^−5^); 2021: CUL (*p* = 3.7 × 10^−7^), 5-hydroxy-CUL: (2.15 × 10^−5^) (15-hydroxy-CUL (*p* = 4.17 × 10^−7^)) (Table S1).

### 2.4. Enniatins and Beauvericin

**Figure 3:**
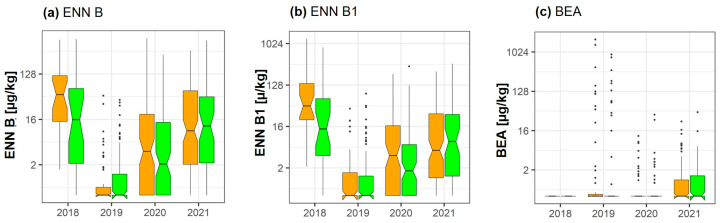
Effect of fungicide treatment on the emerging mycotoxins (**a**) enniatin B (ENN B), (**b**) enniatin B1 (ENN B1), and (**c**) beauvericin (BEA) during the wheat growing seasons in 2018, 2019, 2020, and 2021 in cereal regions in France. Data represent groupwise analysis. Orange bars indicate control samples; green bars indicate fungicide-treated samples.

The effect of fungicide treatment was analyzed with respect to ENN A, A1, and A2, as well as ENN B, B1, and B2. However, only enniatin B and B1 (Figure 3) were present in concentrations exceeding 50 µg/kg at least in one of the four analyzed years. According to the pairwise analysis, significant reductions of ENN B1 (−40.5%) (*p* = 0.043) were observed in 2018, and of ENN B (*p* = 0.022) in 2020 (see supplementary material).

### 2.5. Moniliformin, Aurofusarin, and Rubrofusarin

**Figure 4:**
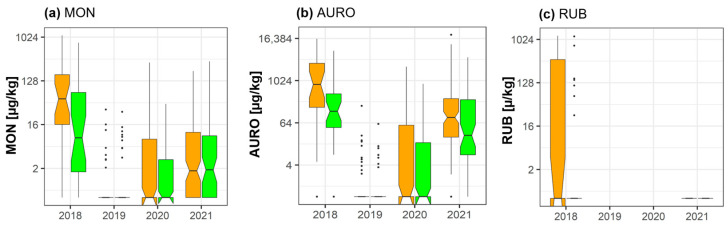
Effect of fungicide treatment on the emerging mycotoxins (**a**) moniliformin (MON), (**b**) aurofusarin (AURO), and (**c**) rubrofusarin (RUB) during the wheat growing seasons in 2018, 2019, 2020, and 2021 in cereal regions in France. Data represent groupwise analysis. Orange bars indicate control samples; green bars indicate fungicide-treated samples.

According to groupwise data analysis, fungicide treatment led to a significant reduction of AURO (−69.8%) (*p* = 0.045) in 2018 (Figure 4). Pairwise data analysis indicated statistically significant reductions for AURO (−69.8%) (*p* = 0.0003) and rubrofusarin (RUB) (−59.3%) (*p* = 0.029) in 2018. Concentrations of AURO varied between the two years, with particularly low levels in 2020 (control = 118 µg/kg) and slightly higher levels in 2021 (control = 799 µg/kg). Statistically significant reductions of mean AURO concentrations were detected in the pairwise data analysis in both years (2020: −64%; *p* = 0.0001; 2021: −55.1%; *p* = 3.7 × 10^−7^).

### 2.6. Alternaria Mycotoxins

**Figure 5:**
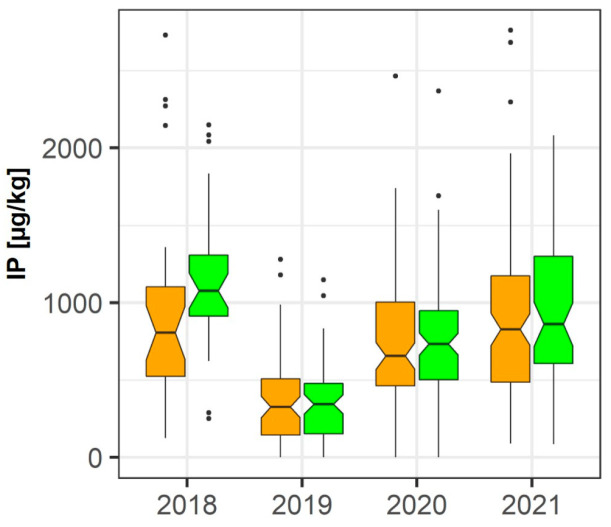
Effect of fungicide treatment on the *Alternaria* toxin infectopyron (IP) during the wheat growing seasons in 2018, 2019, 2020, and 2021 in cereal regions in France. Data represent groupwise analysis. Orange bars indicate control samples; green bars indicate fungicide-treated samples.

*Alternaria*-derived toxins, such as AOH, 4-hydroxy-AOH, AME, Te, or TeA, were either not detected at all or in very low concentrations. Maximum detected levels of these toxins were 0.48 µg/kg (AOH, 2018), 0.05 µg/kg (4-hydroxy-AOH, 2021), 0.23 µg/kg (AME, 2021), 0.42 µg/kg (Te, 2021), and 11 µg/kg (TeA, 2021) in control samples. Consequently, indirect fungicide-induced effects could not be analyzed. Interestingly, however, the *Alternaria*-derived metabolite IP was found in relatively high levels (Figure 5), with mean concentrations of 977 µg/kg in 2018, 349 µg/kg in 2019, 749 µg/kg in 2020, and 894 µg/kg in 2021. Fungicide treatment had either no effect on the levels of IP (2019, 2020) or led to an increase of this metabolite. This increase was statistically significant in 2018 according to the pairwise data analysis scheme (*p* = 0.011) (Table S2).

### 2.7. Butenolide, Tryptophol, and Chrysogin

**Figure 6:**
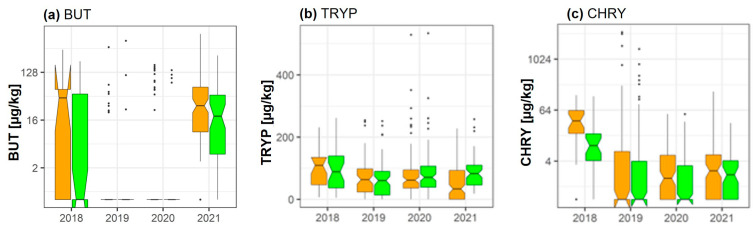
Effect of fungicide treatment on the metabolites (**a**) butenolide (BUT), (**b**) tryptophol (TRYP), and (**c**) chrysogin (CHRY) during the wheat growing seasons in 2018, 2019, 2020, and 2021 in cereal regions in France. Data represent groupwise analysis. Orange bars indicate control samples; green bars indicate fungicide-treated samples.

According to pairwise data analysis, fungicide treatment significantly reduced mean BUT concentrations in 2021 (−52.3%; *p* = 1.14 × 10^−6^). While levels of tryptophol (TRYP) were unaffected by fungicide treatment in 2018, 2019, and 2020, a significant increase (groupwise analysis: *p* = 0.001; pairwise analysis: *p* = 0.01) of the metabolite concentration was seen in 2021 (+72.2%) (Figure 6). According to both the groupwise (*p* = 0.043) and pairwise (*p* = 2.21 × 10^−6^) data analysis scheme, levels of the metabolite chrysogin (CHRY) were significantly reduced by fungicide treatment in 2018 (−57.4%). Furthermore, although present in very low concentrations, CHRY levels were significantly lower in the fungicide treated group in 2020 (−52.3%, *p* = 0.0001) and 2021 (−42.1%, 1.27 × 10^−6^).

### 2.8. Overview of Fungicide Effects

Table 1 shows an overview of the statistically significant effects of fungicide treatments over the 4-year study period. Fungicides induced the strongest effects on the mean concentrations of mycotoxins and fungal metabolites in the year 2018, followed by 2021.

### 2.9. Correlation of Metabolite Occurrence

**Figure 7:**
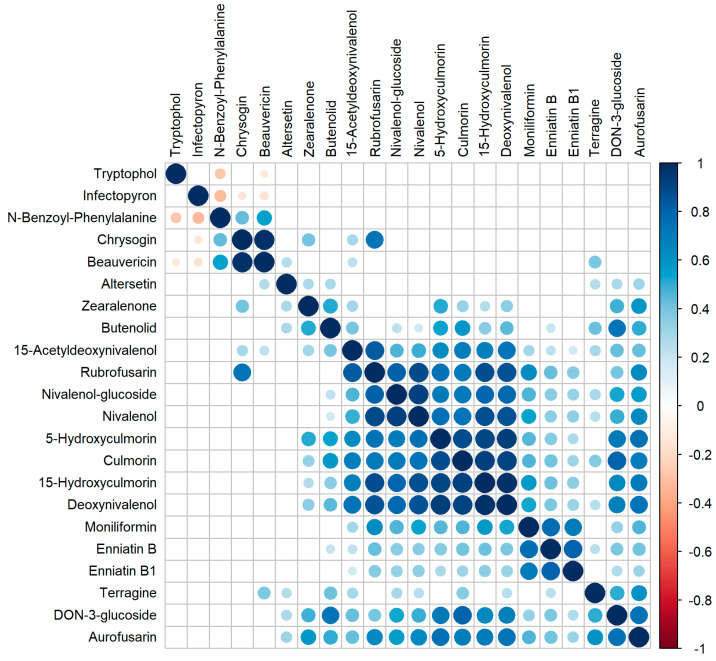
Correlations between major fungal metabolites. Pearson correlation coefficients are shown as colors and size. Non-significant correlation coefficients are shown in white (=same as correlation coefficient is 0). All years of data were taken together. The order of the metabolites was set by default hierarchical clustering as implemented in corrplot.

The entire data set was used to calculate the correlation of mycotoxin concentrations for any combination of two mycotoxins (Figure 7). A strong positive correlation was observed between CHRY and BEA, with a correlation coefficient close to 1. Furthermore, there was a clear positive correlation of the metabolites DON, DON3G, 15ADON, NIV, NIV-3G, RUB, CUL, 5-hydroxy-CUL, and 15-hydroxy-CUL, suggesting a strong degree of co-occurrence of these metabolites. Within this group, a particularly high correlation coefficient can be observed between DON and 15-hydroxyculmorin. Another strong positive correlation can also be seen for the metabolites ENN B, B1, and BEA, as well as for DON3G, terragine, and AURO. Interestingly, a negative correlation was observed between the metabolites infectopyron, N-benzyoyl-phenylalanine, CHRY, and BEA.

## 3. Discussion

The accumulation of mycotoxins in agricultural commodities such as wheat poses a substantial threat not only to human and animal health, but also to the safety of our food supply chain. The metabolites contaminating wheat and other small grains are produced by representatives of various fungal genera, such as *Alternaria*, *Aspergillus*, *Fusarium*, *Claviceps*, and *Penicillium* [63]. In this context, it should also be noted that contamination with single mycotoxins is rare—instead, co-contamination is mostly the rule [15,29]. In addition to the “traditional” mycotoxins, it is particularly important to consider the emerging and masked mycotoxins and the fact that their co-occurrence may result in negative effects, due to their potential additive and/or synergistic effects [14]. According to Gruber-Dorninger et al., 2019 [2], for example, at least two or more mycotoxins were found in 64% of over 70,000 analyzed feed samples, including maize, wheat, and soybeans from 100 countries. Furthermore, a Spanish study has shown that 77% of barley samples were contaminated with two or more mycotoxins belonging to the type A and type B trichothecenes [64]. Thus, investigations focusing on the occurrence and co-occurrence of such fungal-derived toxins, as well as suitable pre- and post-harvest mitigation strategies aiming to reduce contamination levels and therefore the detrimental health effects for both humans and animals, are essential. Our study not only provides a 4-year investigation of the simultaneous occurrence of an exceptionally large number of fungal metabolites in French wheat samples, but we also evaluated the efficacy of fungicide treatment with respect to mycotoxin contamination levels.

Considering all four sampling years (2018–2021), the most abundant mycotoxins were derived from *Fusarium* strains, including DON (970 µg/kg), CUL (889 µg/kg), 15-hydroxy-CUL (780 µg/kg), aurofusarin (586 µg/kg), 5-hydroxy-CUL (498 µg/kg), NIV (112 µg/kg), and DON3G (92 µg/kg). In fact, with regard to their occurrence, strong positive correlations were found between *Fusarium* metabolites, including DON, CUL, 5- and 15-hydroxy-CUL, NIV, NIVG, RUB, 15ADON, DON3G, BUT, MON, ENN B and B1, and AURO. Infestation of crops with *Fusarium* species, such as *F. avenaceum*, *F. graminearum*, *F. culmorum*, and *Microdochium nivale*, often results in Fusarium head blight (FHB), one of the most common and concerning diseases of small-grain cereals. FHB epidemics have been found in all large grain-growing regions worldwide, including China [65,66], South America [67,68,69,70], India and Pakistan [71], the United States [60,72], the Orange River valley of South Africa [73], Canada [74,75], northern and central Europe [76], and Australia.

In addition to the above-mentioned Fusarium metabolites, the results of the current study reveal particularly high levels of the less well-known and only scarcely described *Alternaria* toxin infectopyron (IP), which was present at an average concentration of 733 µg/kg in untreated wheat samples between 2018 and 2021. Interestingly, a similar finding was published in a recent investigation of the occurrence of fungal metabolites in different winter wheat varieties in Croatia. The study reported high levels of IP (approx. 600 µg/kg) in naturally infected wheat varieties [77]. Furthermore, a large-scale analysis of fungal metabolites in grain and straw samples of barley in Switzerland, conducted in the crop seasons of 2016 and 2017, also confirmed the presence of infectopyron in over 50% of samples, exceeding concentrations of 1000 µg/kg [78]. Other metabolites which were present quite abundantly in the current study were terragine (527 µg/kg) and tryptophol (70 µg/kg). Similarly high levels of the latter metabolite (40–60 µg/kg) were also reported by Spanic et al., 2020 [77]. Thus, the current study underlines the global concern which arises from the existence of well-known mycotoxins on the one hand, but also from high levels of unregulated metabolites on the other hand. In particular, the latter aspect urgently calls for further research on occurrence and toxicology.

Nevertheless, regardless of the current availability of information on mycotoxins or regulation of mycotoxin levels in cereals, mitigation strategies should be implemented at pre- and post-harvest stages. As the contamination of crops begins in the field, it is particularly important to address this problem at the earliest possible stage. Preharvest mitigation strategies are essential tools to avoid fungal infestation, thereby minimizing the resulting level of mycotoxin contamination during later stages of harvest and storage. With respect to wheat, pre-harvest crop management must cover several aspects, such as cultivar resistance, the use of fungicide and/or biocontrol agents, suitable planting, and harvest times, as well as practices such as crop rotation, tillage, and fertilization. These mitigation strategies aim to decrease the amount of inoculum in the field, inhibit plant infection at flowering, and reduce disease spread within wheat ears [63].

The current study underlines the effectiveness of fungicide treatment, especially with respect to reducing contamination with the trichothecenes DON, DON3G, and NIV, as well as CUL and its derivatives, 5-hydroxy-CUL and 15-hydroxy-CUL, and AURO. All these metabolites were significantly reduced as a result of fungicide treatment in the years 2018, 2020, and 2021 according to both or at least one of the data analysis schemes (Table 1).

Interestingly, the fungicide-induced decrease of *Fusarium* metabolites was accompanied by a significant increase of the *Alternaria* metabolite IP and the metabolite TRYP (Figure 7). Furthermore, correlation analysis indicated a negative correlation of both IP and TRYP concentrations with concentrations of *Fusarium* metabolite BEA and *Penicillium* metabolite N-benzoyl-phenylalanine (Figure 7). Similarly, Drakopoulos et al., 2021 [78] reported a negative correlation between the occurrence of IP and a number of *Fusarium* metabolites (e.g., AURO, CUL, 5-hydroxy-CUL, 15-hydroxy-CUL, DON, ENNs, MON, ZEN, …) in the grain and straw of barley samples. A negative correlation between metabolites produced by *Fusarium* spp., *Alternaria* spp., and *Penicillium* spp. could be due to competition between these fungi. This hypothesis is supported by the results of a recent study, which found a reduction of the concentration of Alternaria toxins, including IP, and the metabolite TRYP, following *Fusarium* inoculation of wheat [77]. It could be interesting to further investigate the effect of fungicide treatment on the ecological relationship between different fungal populations in future studies.

There was a strong variation of contamination levels noticeable over the 4-year study period, with relatively high contamination levels in 2018 and 2021 and far lower contamination levels in 2019 and 2020. Studies have reported that year-to-year fluctuations of mycotoxin contamination levels are not uncommon and are often a result of varying physical or chemical factors which either favor or limit mycotoxin occurrence (e.g., moisture, relative humidity, temperature, stress, etc.) [79,80].

While the data presented here support the efficacy of fungicide treatment to reduce concentrations of major mycotoxins of concern such as DON in wheat, complete prevention of mycotoxin contamination was not achieved. For example, despite a significant fungicide-induced reduction of DON concentrations in 2018, the year with the highest contamination level in our dataset, a mean DON concentration of 2434 µg/kg remained in the wheat samples (Figure 1). In 2019, the year of the lowest overall contamination, DON concentrations were unaffected by fungicide treatment and a mean concentration of 244 µg/kg DON remained in the treated wheat sample. In 2020 and 2021, fungicide treatment was effective, leading to significant reductions of DON. Nevertheless, concentrations of 110 µg/kg (2020) and 356 µg/kg (2021) were still detected in the fungicide-treated samples. Furthermore, it should be considered that even low concentrations of fungal-derived toxins remaining in the samples cannot be neglected. The potential and mostly unpredictable synergistic, additive, or antagonistic interactions among mycotoxins can lead to severe health problems for both humans and animals, even if the concentration of single metabolites are below regulatory guidelines [81]. In this context, it is interesting to mention that due to increasing evidence regarding the risk of even low mycotoxin concentrations, the European Food Safety Authority (EFSA) has only recently initiated assessments regarding the no-observed-adverse-effect levels (NOAEL) of mycotoxins such as DON and FUM in several species [82,83].

While the use of fungicides presents one essential tool to reduce mycotoxin contamination of agricultural commodities, it is also—in this context—important to address the aspect of residual fungicides and possible degradation products left in the environment and their effects on human and animal health. This problem highlights the importance of respective research focusing on the attenuation of the toxicity and occurrence of degradation products of fungicides, as well as the usage of alternative pre-harvest strategies. One such example was recently published by Del Puerto et al., 2022 [84], showing the positive effects of combined vacuum UV and UVC treatment to reduce the toxicity and occurrence of degradation products of the fungicide tebuconazole in drinking water.

Thus, while the current study clearly confirms the importance of fungicide application in the field as a pre-harvest mitigation strategy, it not only highlights the necessity of counteractive strategies to minimize detrimental effects of potential chemical residues, but also underlines the fact that the effective management of mycotoxin contamination in wheat and other agricultural commodities cannot be based on this mitigation measure alone. Instead, fungicide treatment in the field must present one important mitigation tool within an array of different pre- and post-harvest approaches to effectively reduce mycotoxin contamination and improve food and feed safety. These include early in-field management techniques, such as crop selection [40,41,42,43,44,45,46,47,48,49], crop rotation [50,51,52], tillage [53], and fertilization [54], and secondary techniques such as careful timing of planting and harvest times. In addition, postharvest techniques including suitable storage conditions and moisture adjustment [32,33], as well as the use of feed additives that enable biodegradation [34,35,36] or adsorption [37,38,85] of fungal-derived metabolites, must be part of an integrated mitigation strategy to reduce levels of fungal-derived metabolites to a minimum.

## 4. Materials and Methods

### 4.1. Experimental Design

The experiments were conducted during the wheat growing seasons in 2018, 2019, 2020, and 2021 in cereal regions in France. Common wheat and durum wheat were used for the study, which are both commercially available and widely used by farmers throughout France.

### 4.2. Fungicide Application

In 2018, prothioconazole-containing fungicides (Prosaro and Kestrel [both: 0.6–1.2 L/ha]) were applied. In 2019, 2020, and 2021, the prothioconazole-containing commercially available fungicides Fandango (1.2 L/ha) and Madison (0.7 L/ha) were applied. The fungicides are currently distributed by Bayer AG and were applied to the wheat via spraying from the beginning until the end of flowering. Real crop fields distributed all over France were split into two separate areas. Fungicides were applied to one area once at the beginning of flowering, while the other area (control) remained untreated. Samples were collected at grain maturity, just prior to harvesting. In each instance, one sample was taken from the control and treated fields. The sample size was 3 kg of grain or 600 ears in each strip. The total number of collected wheat samples amounted to 59 in 2018, 139 in 2019, 193 in 2020, and 171 in 2021.

### 4.3. Quantification of Mycotoxins via LC-MS/MS Multi-Analyte Method

All samples were analyzed for the presence and concentration of fungal metabolites via LC-MS/MS spectrometry according to the method of Sulyok et al., 2020 [86]. Briefly, samples were delivered to the Institute of Bioanalytics and Agro-Metabolomics at the University of Natural Resources and Life Sciences Vienna (BOKU) in Tulln, Austria. A ground sample aliquot of 5 g was extracted with a mixture of 20 mL acetonitrile, water, and acetic acid (79:20:1, per volume) on a rotary vapor for 90 min. Samples were then centrifuged, and the supernatant was subsequently transferred to glass vials and diluted 1:1 with a mixture of acetonitrile, water, and acetic acid (20:79:1, per volume). The samples were injected into the LC-MS/MS system using electrospray ionization and mass spectrometric detection via a quadrupole mass filter. Quantification was performed according to an external calibration using a multi-analyte stock solution.

### 4.4. Statistical Analysis

#### 4.4.1. Filtering and Processing

For analysis per year, no filtering was performed. For the comparisons, when merging all years together, only those metabolites were taken into account which were measured at least 150 times, and which showed measured values > LOQ more than 5 times. Of these metabolites, all values were taken into account. A few more metabolites were removed from comparison because they did not have enough different values for a statistical comparison test. Table S2 provides a list of metabolites with enough data.

Samples were split into control and treatment groups—all treatments were merged into one group. If more than one treatment was applied at the same field, one of them was selected randomly for analysis. There were 25 potentially different treatments applied.

#### 4.4.2. Clustering

Investigation for potential effects was carried out visually via principal component analysis (PCA) (Figures S1–S45) and hierarchical clustering. For PCA, the built-in function prcomp in R [87] was used. Hierarchical clustering was performed with the R package ape [88].

#### 4.4.3. Differences between Control and Treatment

The significance of metabolite levels difference between treatments—control vs. treatment—was determined with Wilcoxon tests, and additionally with a *t*-test if the null hypotheses of a normal distribution was not rejected in both of the two groups. The significance of a non-normal distribution was assessed with Kolmogorov–Smirnov tests (function ks.test in R).

When metabolites were analyzed together, their values were summed up.

The differences between treatments were tested per metabolite groupwise and pairwise. For the groupwise analysis, all values of controls were taken together and compared with all values of the treatments. It was tested whether the median or mean was different in these two distributions.

For the pairwise analysis, the pairwise differences between treatment and control were taken. Data in which only control or treatment were present, were neglected. It was tested whether the median or mean difference was significantly different from zero.

Results of groupwise analysis are shown within the section ‘Results’ and compared with results of the pairwise analysis. Data of the pairwise analysis are presented in the supplementary section (Table S1).

#### 4.4.4. Visualization

Beside the standard R functions, the package corrplot [89] in R was used for visualization.

## Figures and Tables

**Table 1:** Overview of the statistically significant effects of fungicide treatment on the mean concentration of mycotoxins and metabolites over the 4-year study period (2018–2021). Green arrows pointing downwards indicate a statistically significant decrease in the mean concentration. Red arrows pointing upwards indicate a statistically significant increase in the mean concentration (GW = groupwise analysis; PW = pairwise analysis).

	2018		2019		2020		2021	
Metabolites	GW	PW	GW	PW	GW	PW	GW	PW
DON	**🡫** −59.8% (*p* = 0.044)	**🡫** −59.8% (*p* = 5.51 × 10^−6^)				**🡫** −65.4% (*p* = 4.13 × 10^−5^)		**🡫** −47.1% (*p* = 3.7 × 10^−7^)
DON3G	**🡫** −53.9% (*p* = 0.044)	**🡫** −53.9% (*p* = 0.00031)				**🡫** −61.7% (*p* = 0.0002)		**🡫** −18.2% (*p* = 1.67 × 10^−7^)
15-ADON		**🡫** −71% (*p* = 0.048)						**🡫** −49.6% (*p* = 0.0009)
NIV	**🡫** −59.1% (*p* = 0.044)	**🡫** −59.1% *p* = 0.00031				**🡫** −58.3% (*p* = 0.006)		**🡫** −15.6% (*p* = 0.019)
NIV-G	**🡫** −57% (*p* = 0.044)	**🡫** −57% (*p* = 0.002)						
CUL	**🡫** −54.2% (*p* = 0.046)	**🡫** −54.2% (*p* = 0.0005)				**🡫** −64.5% (*p* = 0.0002)		**🡫** −40.3% (*p* = 3.7 × 10^−7^)
5-hydroxy-CUL	**🡫** −62% (*p* = 0.044)	**🡫** −62% (*p* = 0.0003)				**🡫** −74% (*p* = 0.0005)		**🡫** −39.8% (*p* = 2.13 × 10^−5^)
15-hydroxy-CUL	**🡫** −57.3% (*p* = 0.044)	**🡫** −57.3% (*p* = 0.00003)				**🡫** −64.4% (*p* = 4.13 × 10^−5^)		**🡫** −45.5% (*p* = 4.17 × 10^−7^)
ENN B						**🡫** −62.4% (*p* = 0.022)		
ENN B1		**🡫** −40.5% (*p* = 0.043)						
BEA								
MON								
AURO	**🡫** −69.8% (*p* = 0.045)	**🡫** −69.8% (*p* = 0.0003)				**🡫** −64.3% (*p* = 0.0001)		**🡫** −55.1% (*p* = 3.7 × 10^−7^)
RUB		**🡫** −59.3% (*p* = 0.029)						
IP		**🡩** +19.8% (*p* = 0.011)						
BUT								**🡫** −47.7% (*p* = 1.14 × 10^−6^)
TRYP							**🡩** +72.2% (*p* = 0.001)	**🡩** +72.2% (*p* = 0.01)
CHRY	**🡫** −57.5% (*p* = 0.043)	**🡫** −57.5% (*p* = 2.21 × 10^−6^)						

## Data Availability

Data is contained within the article of Supplementary Material.

## Supplementary Materials

The following supporting information can be downloaded at: https://www.mdpi.com/article/10.3390/toxins15070443/s1, Figures S1–S45: PCA and hierarchical clustering for the investigation of potential biases of individual fungicides; Table S1: Effect of fungicide treatment on mycotoxins and fungal metabolites according to the pairwise data analysis scheme.; Table S2: Complete list of fungal metabolites included in the filtering and processing analysis per year.
