# Supplementary figures and images for: Effect of Fungicide Treatment on Multi-Mycotoxin Occurrence in French Wheat during a 4-Year Period
Source: Toxins (Basel). 2023 Jul 4;15(7):443. doi: 10.3390/toxins15070443 (PMC10467151; doi:10.3390/toxins15070443)

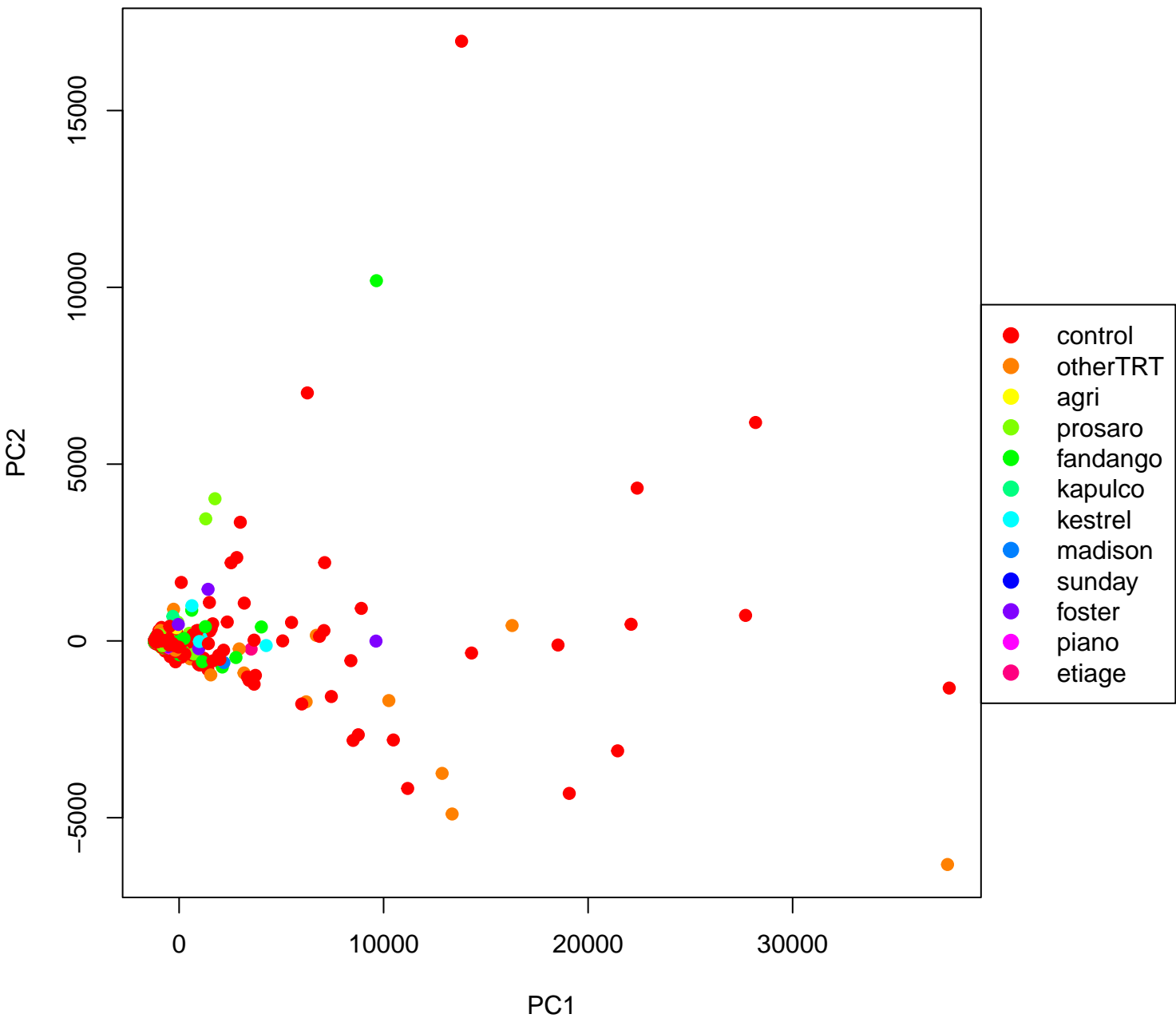

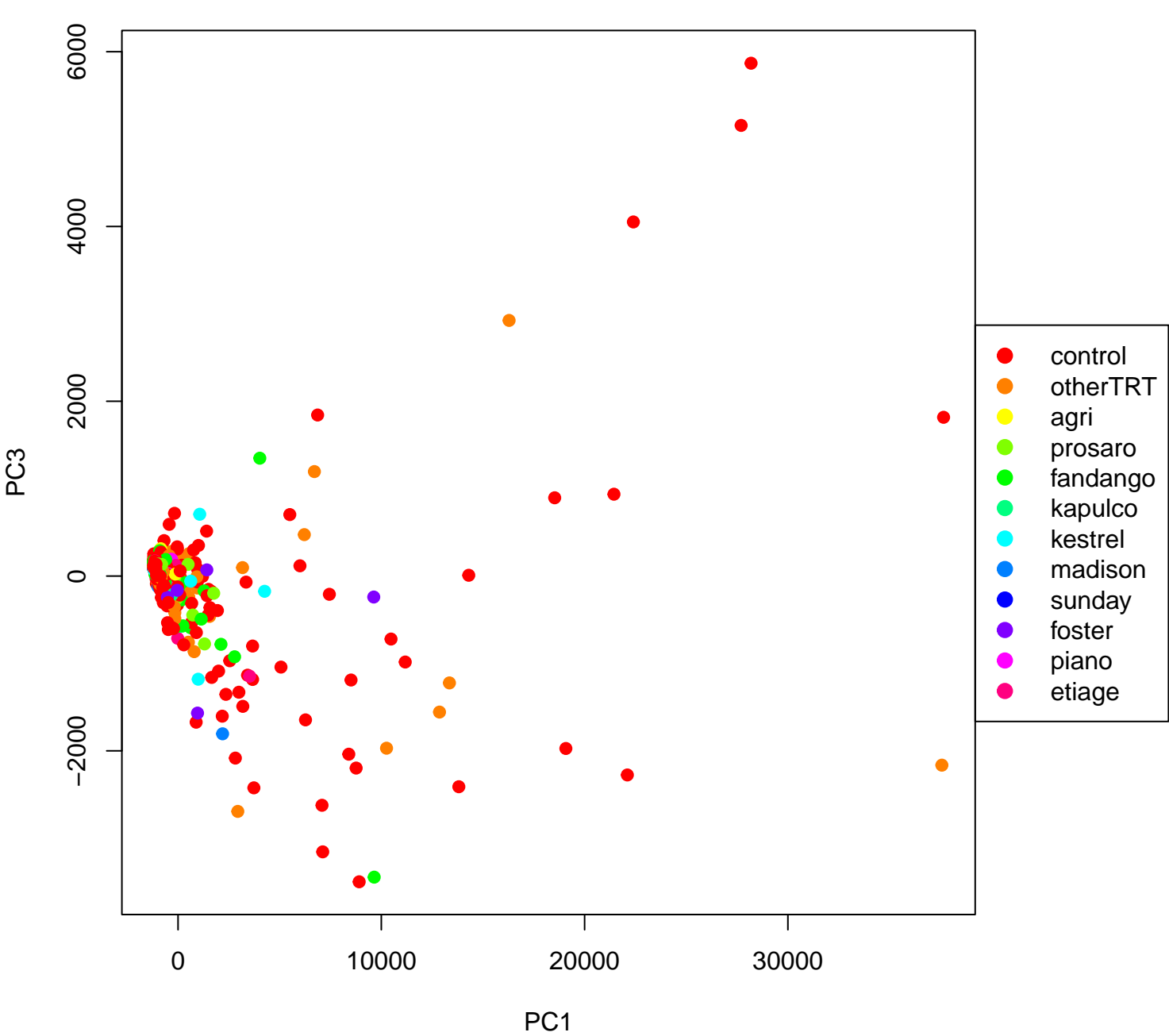

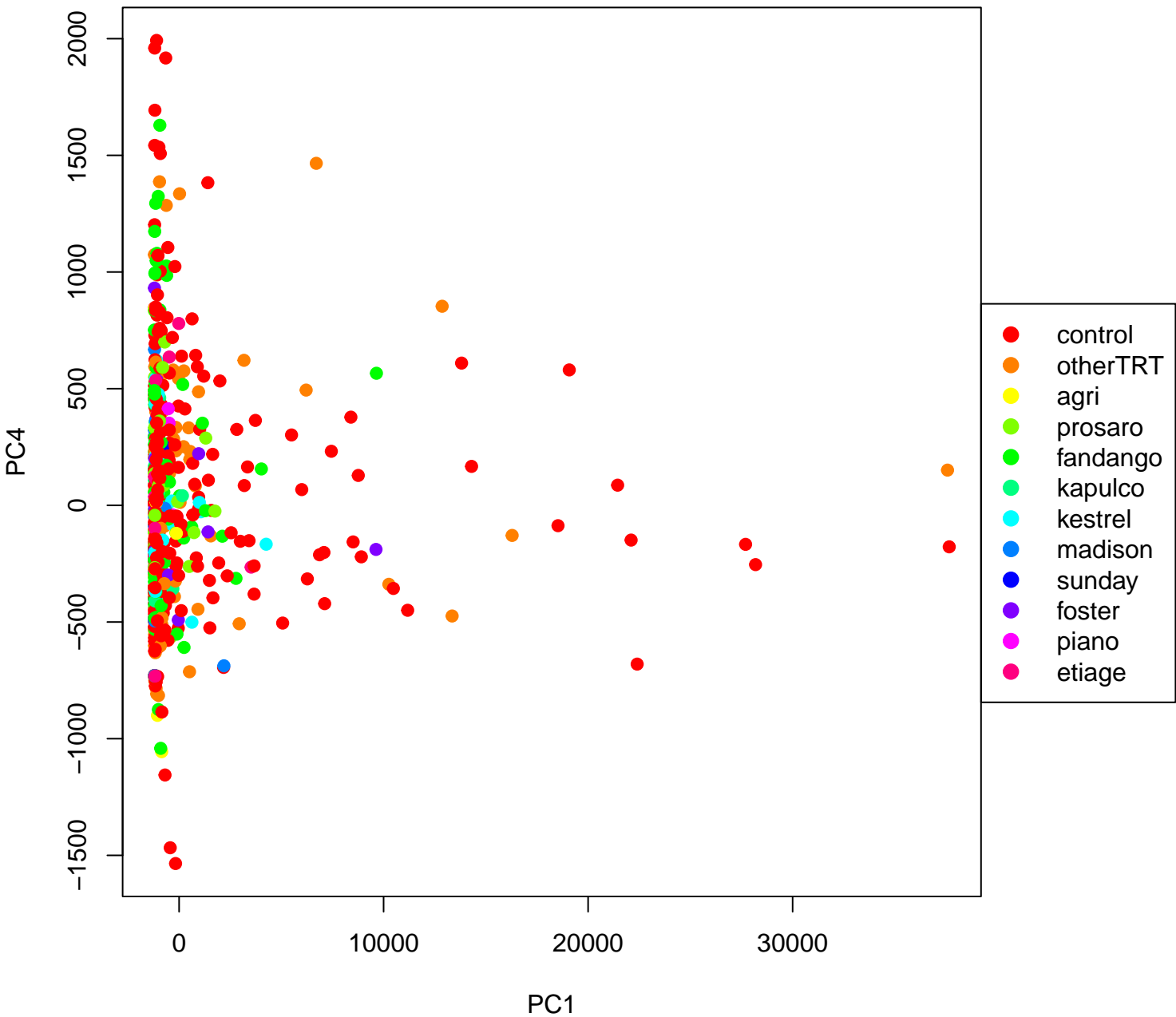

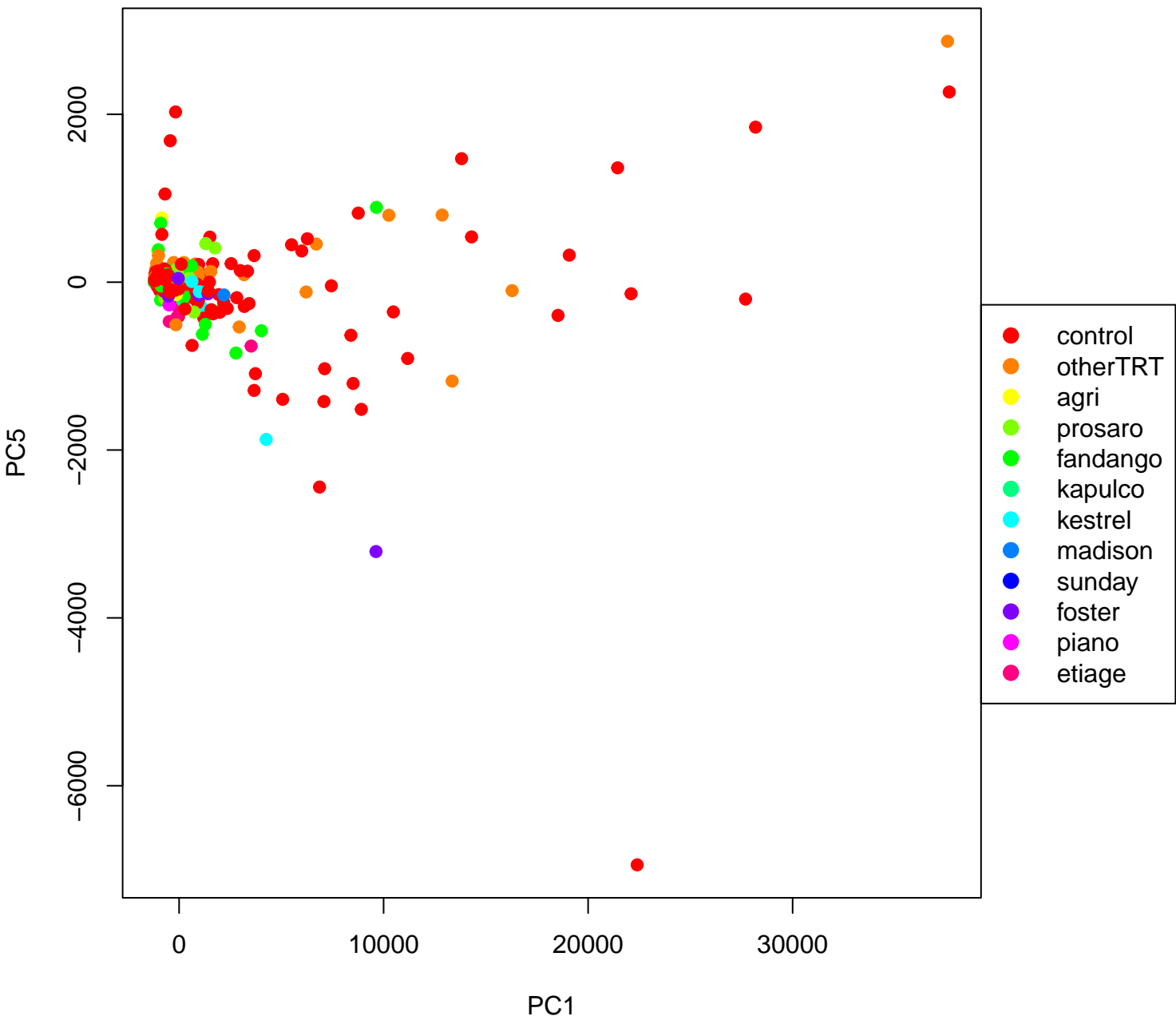

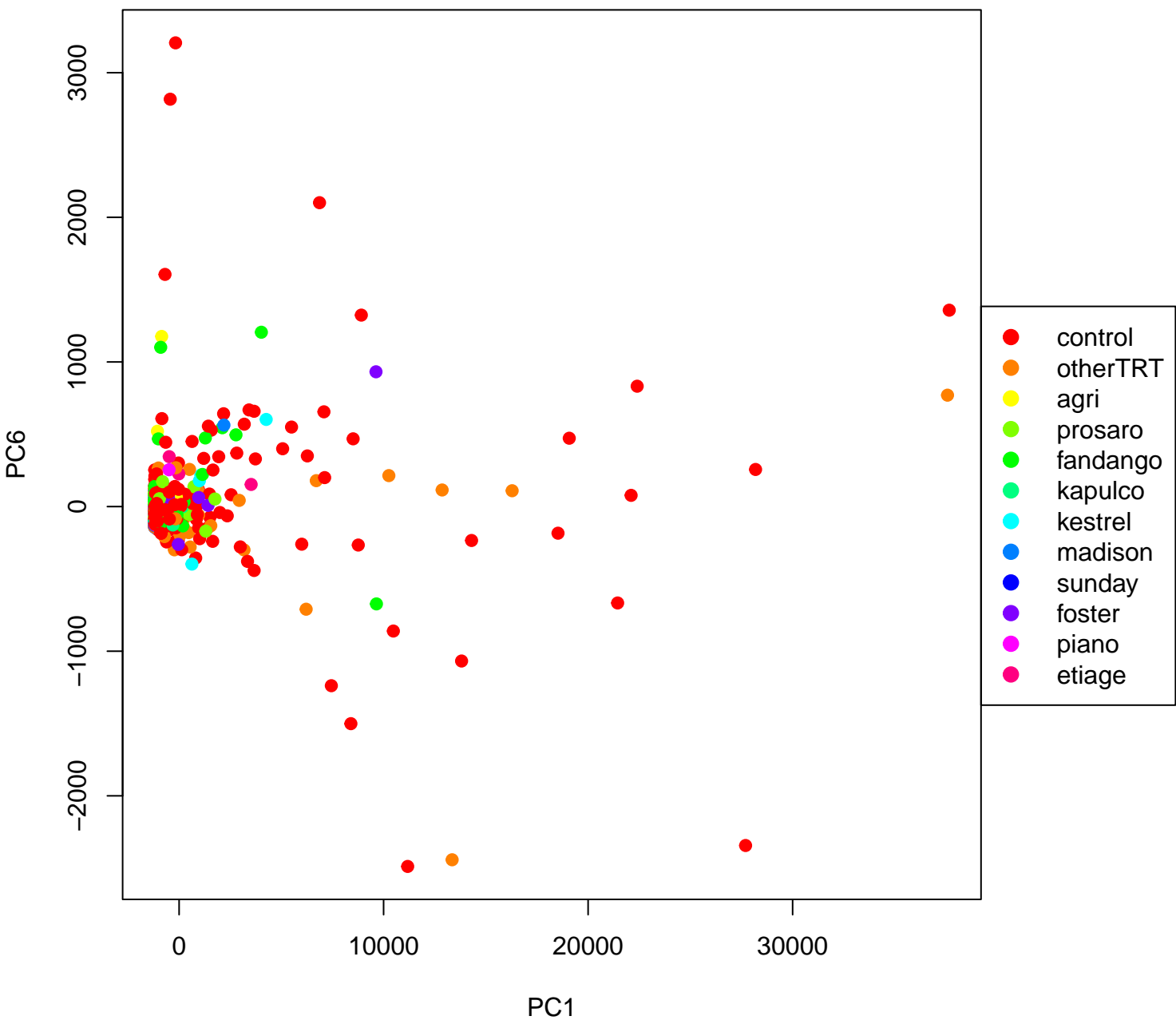

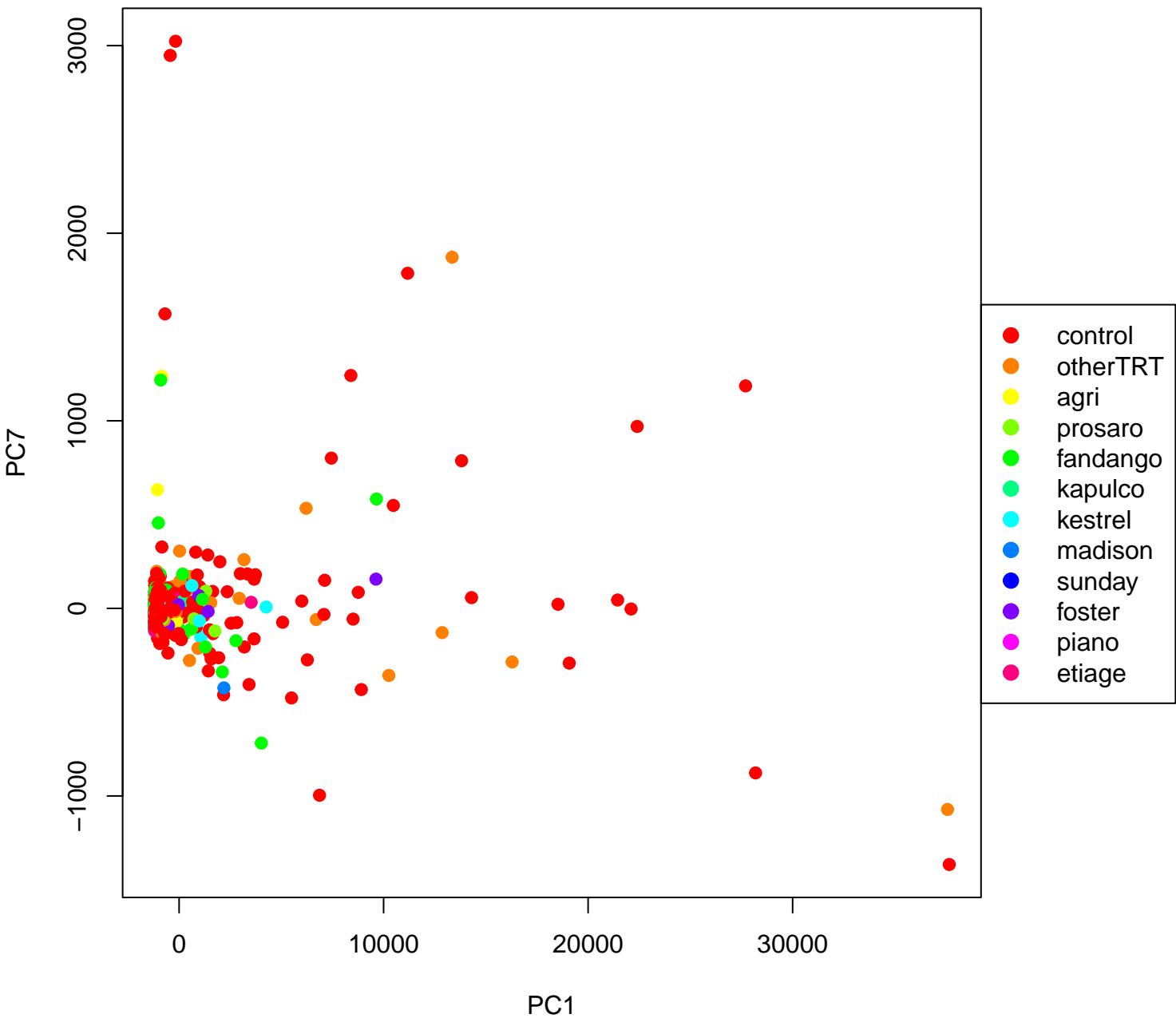

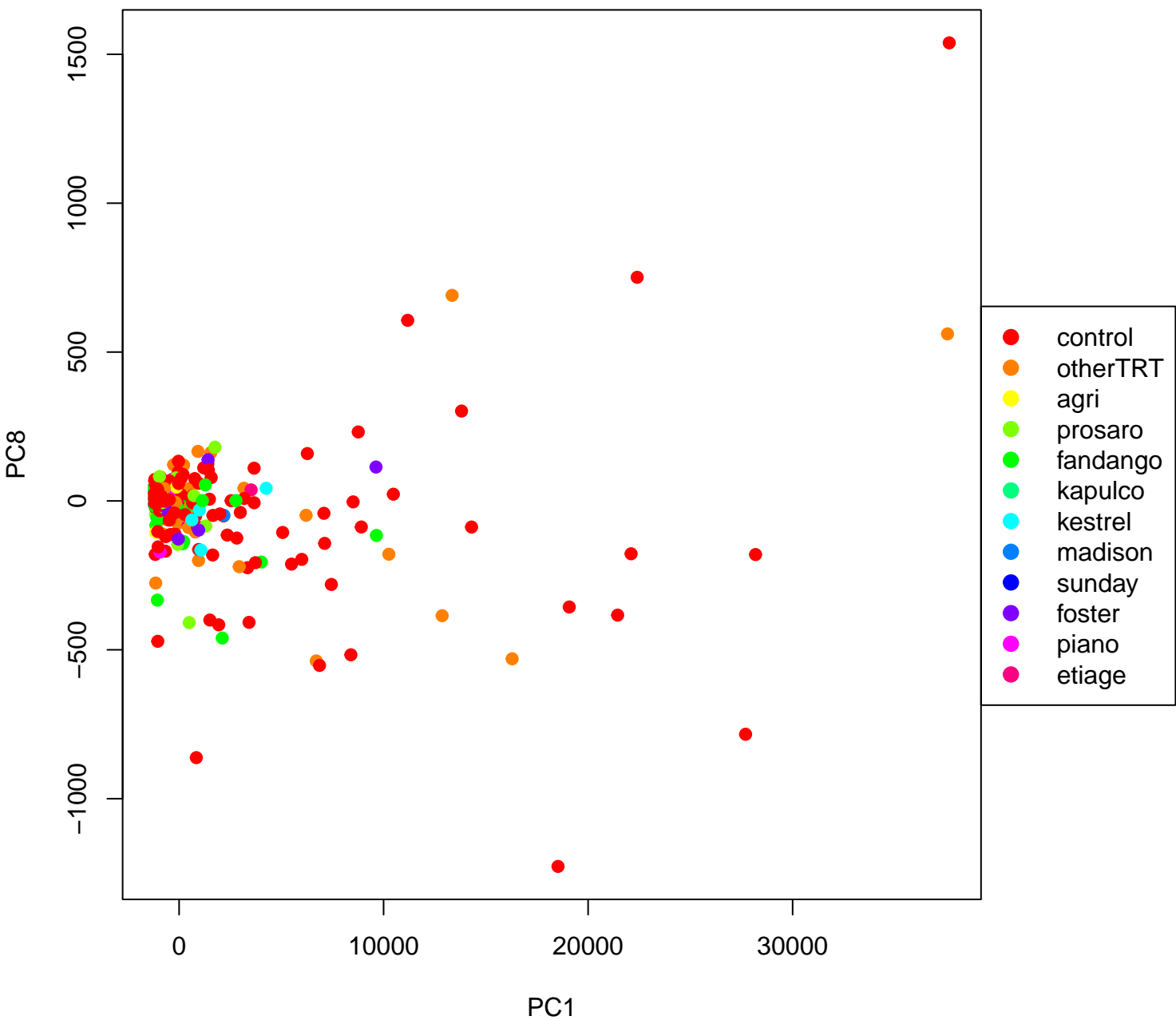

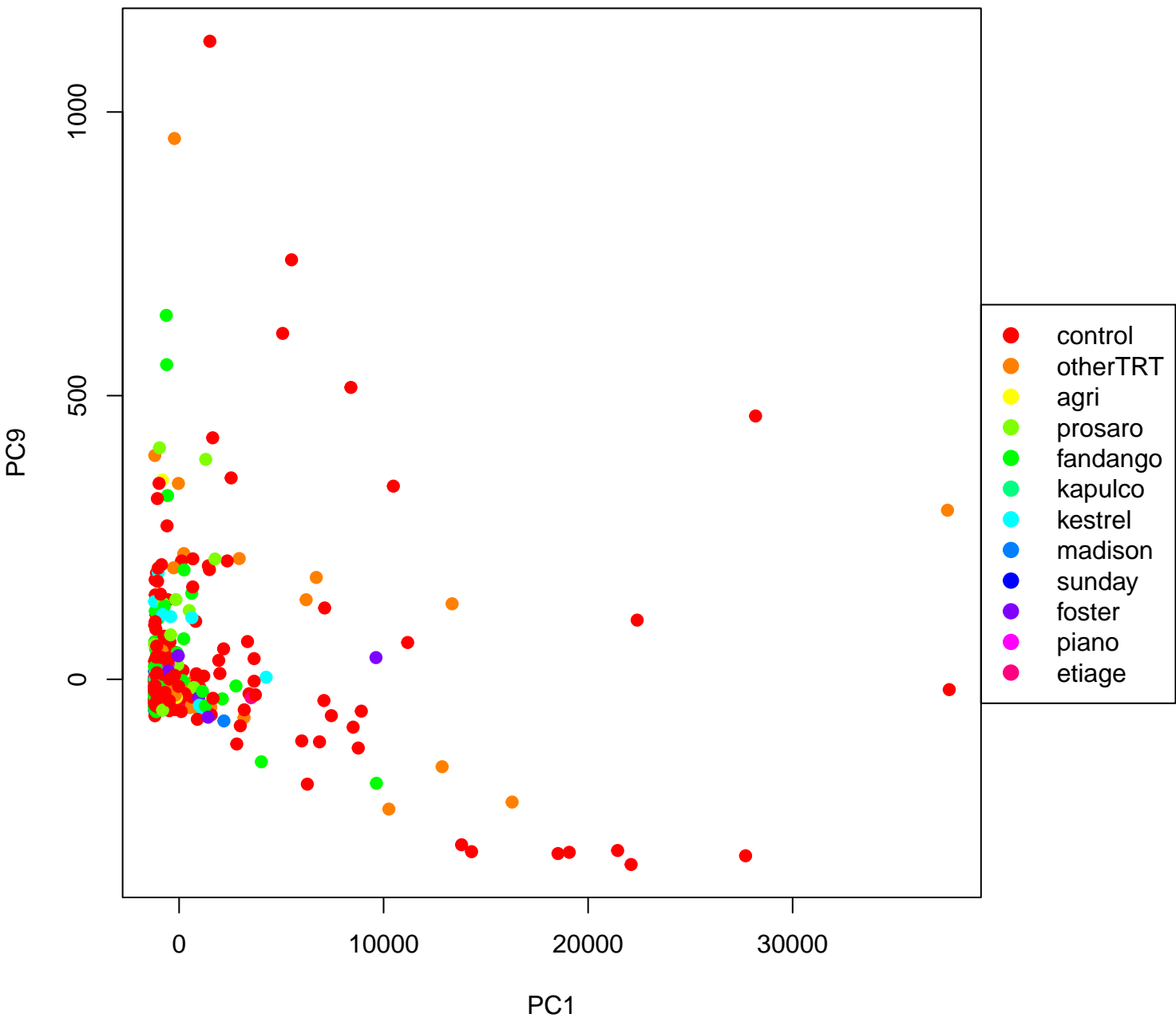

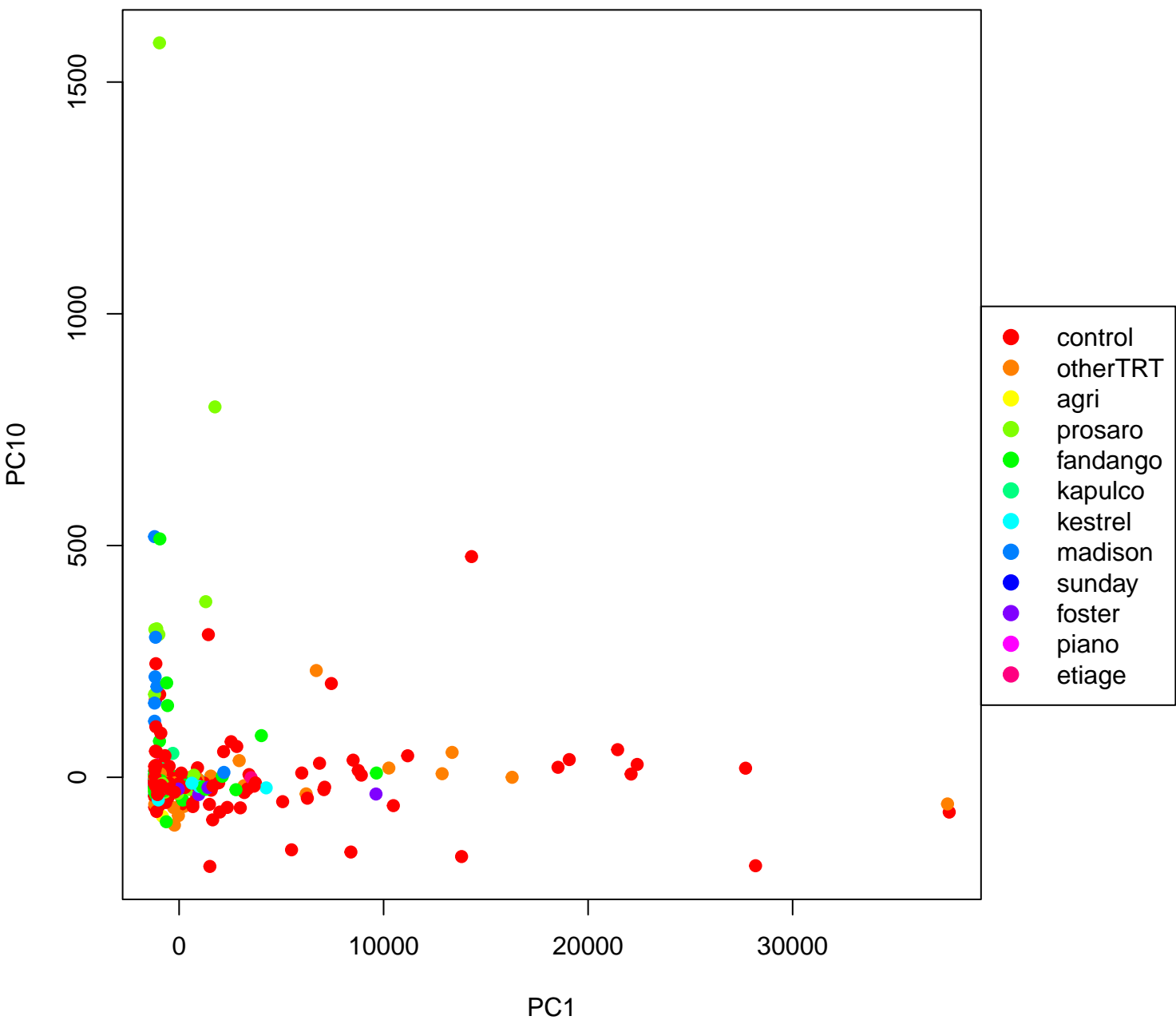

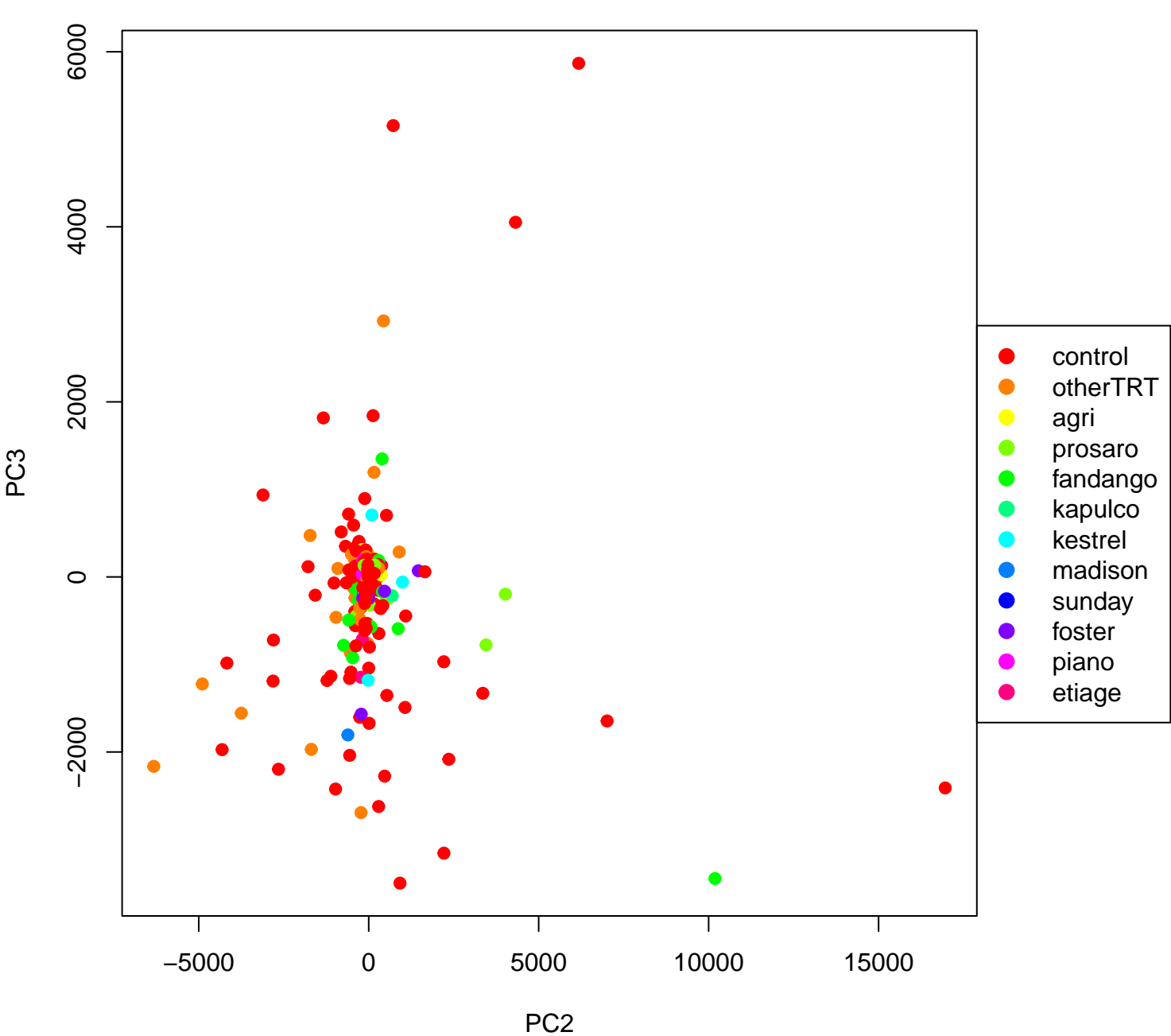

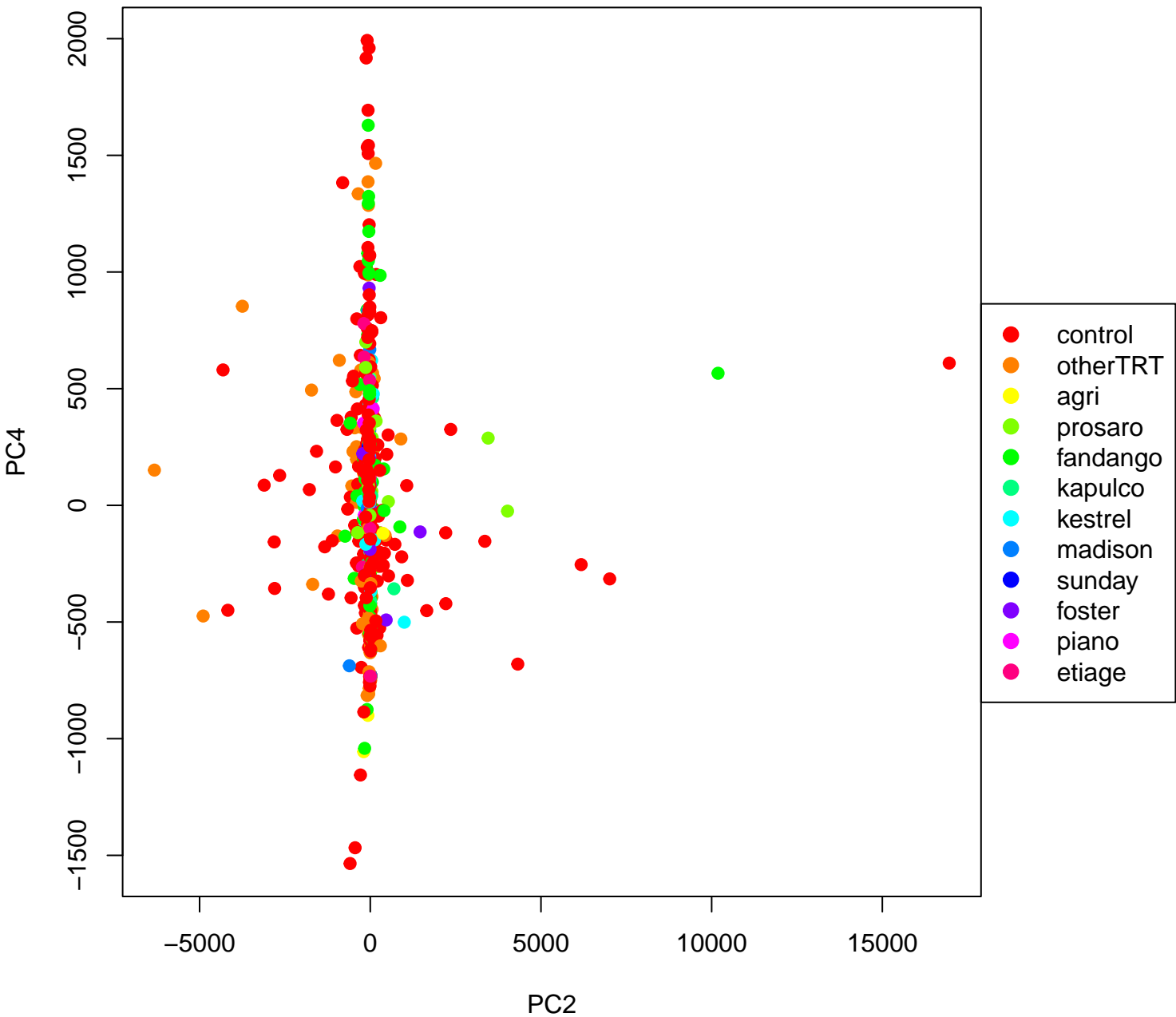

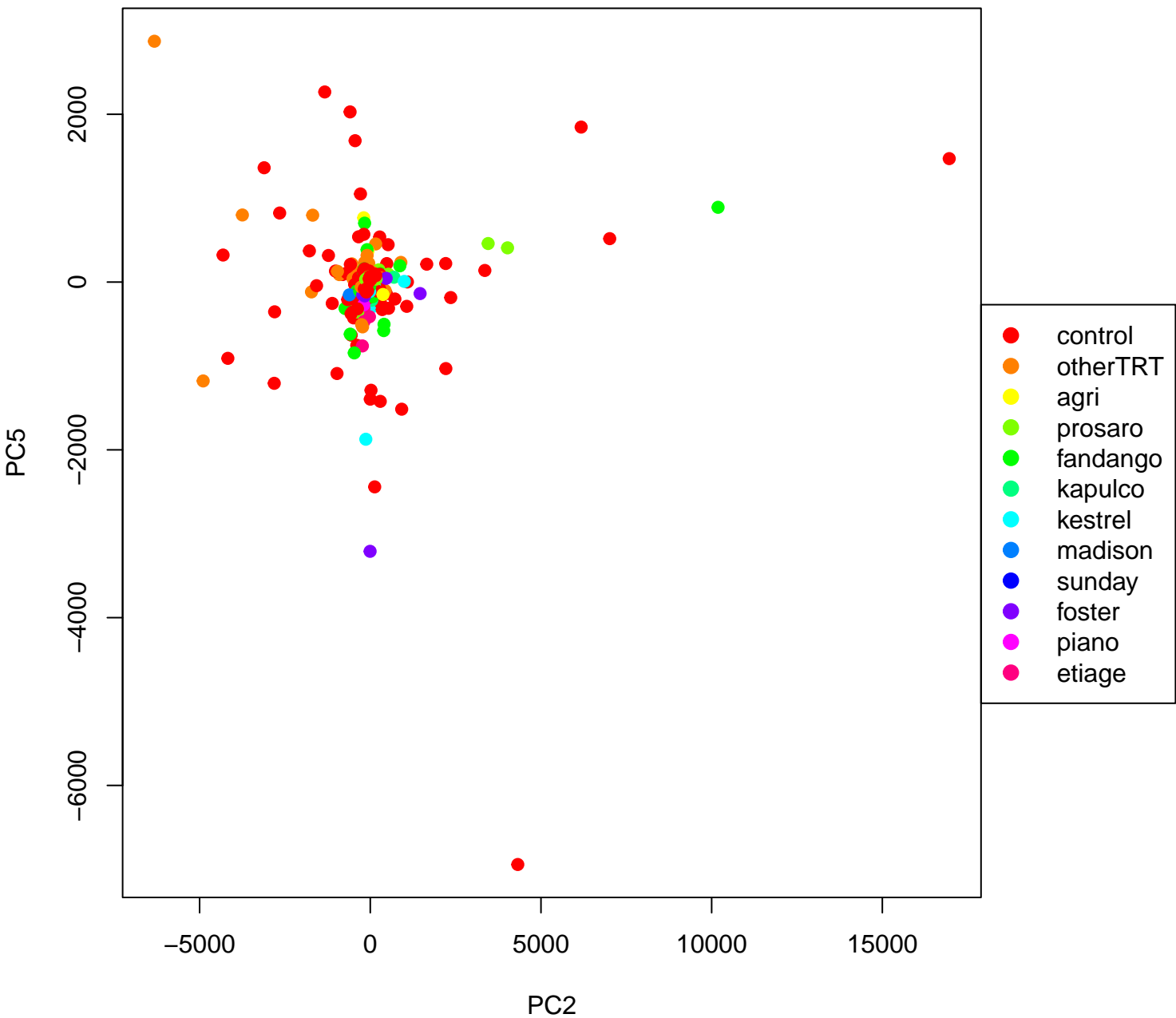

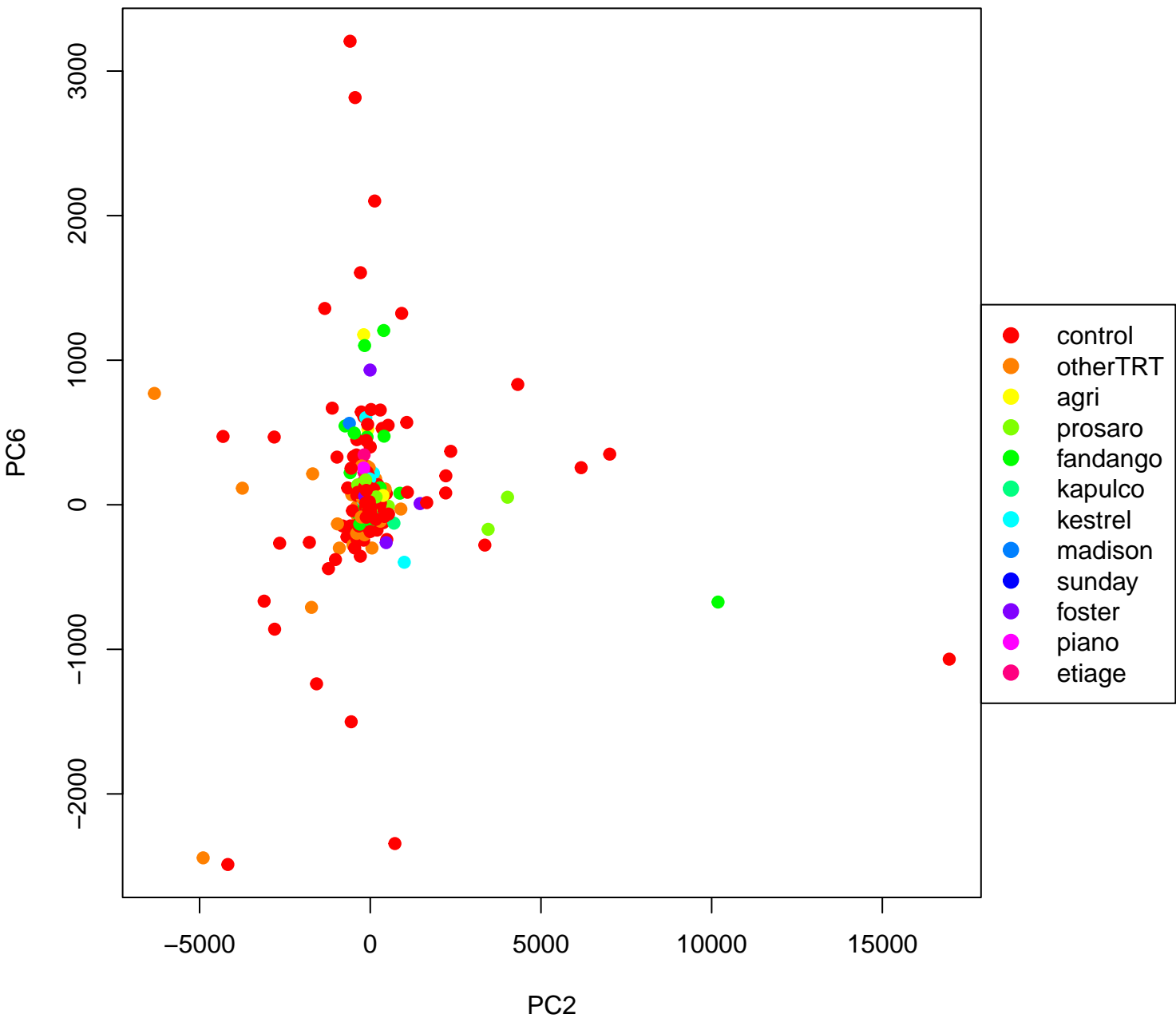

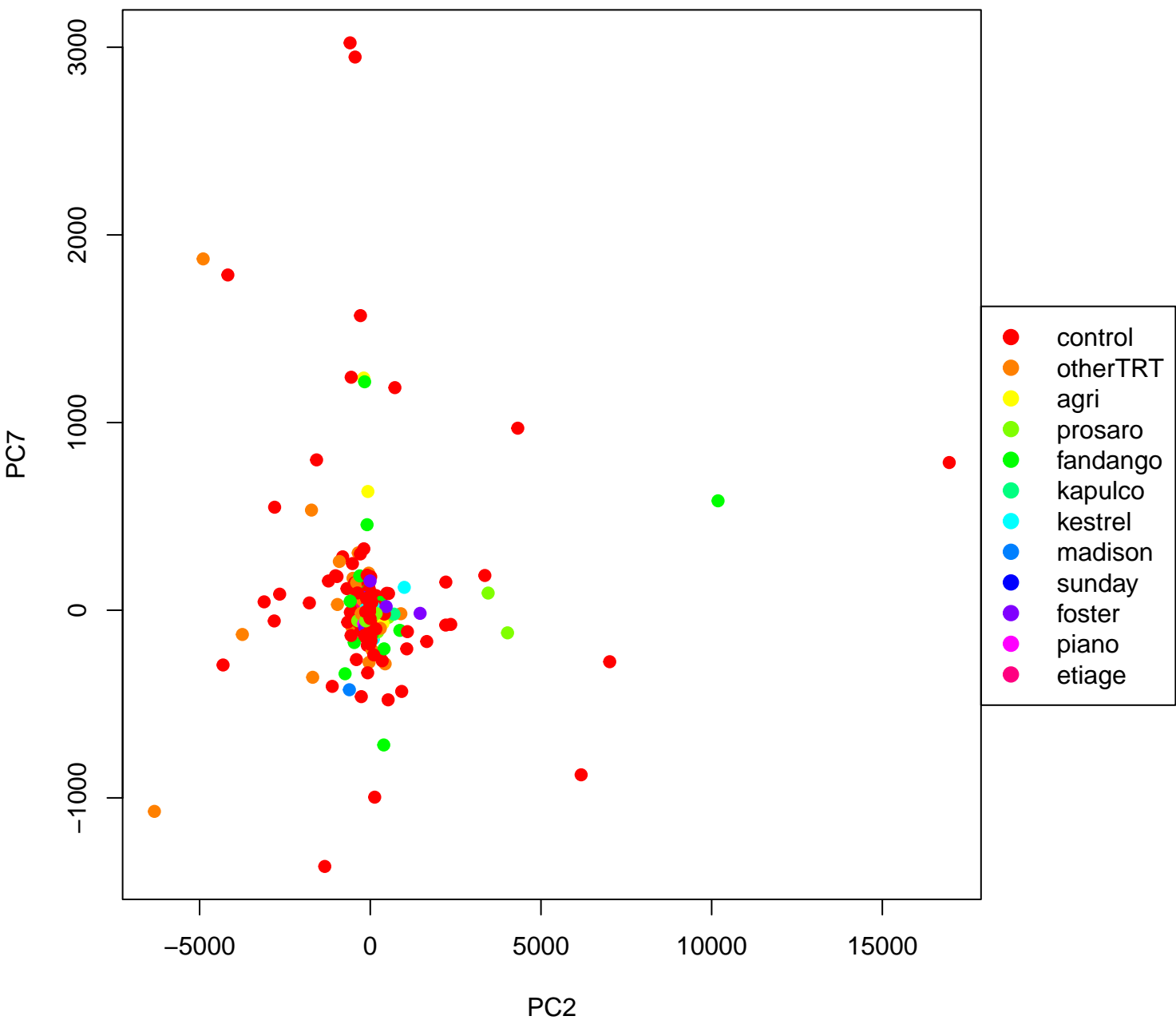

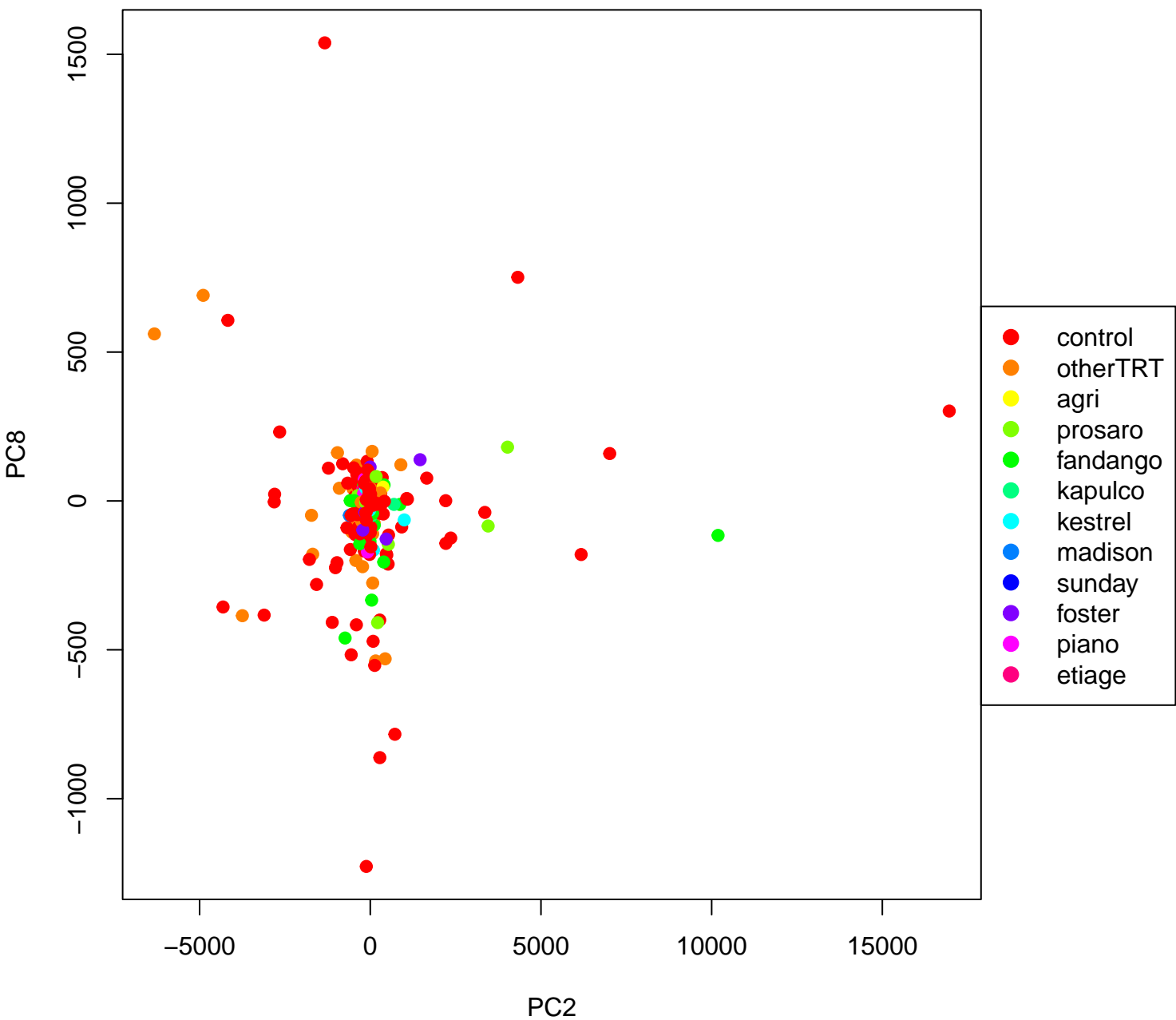

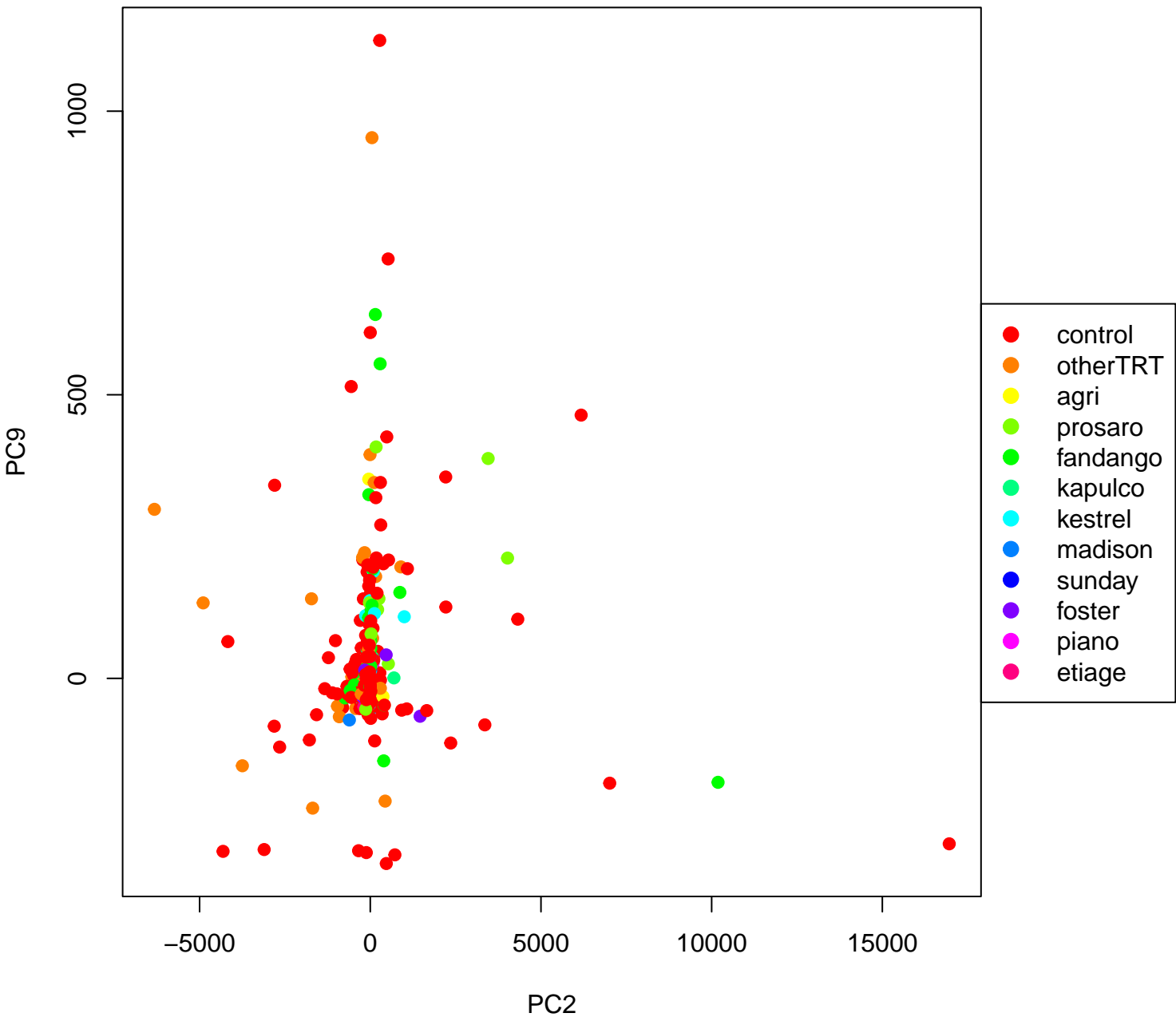

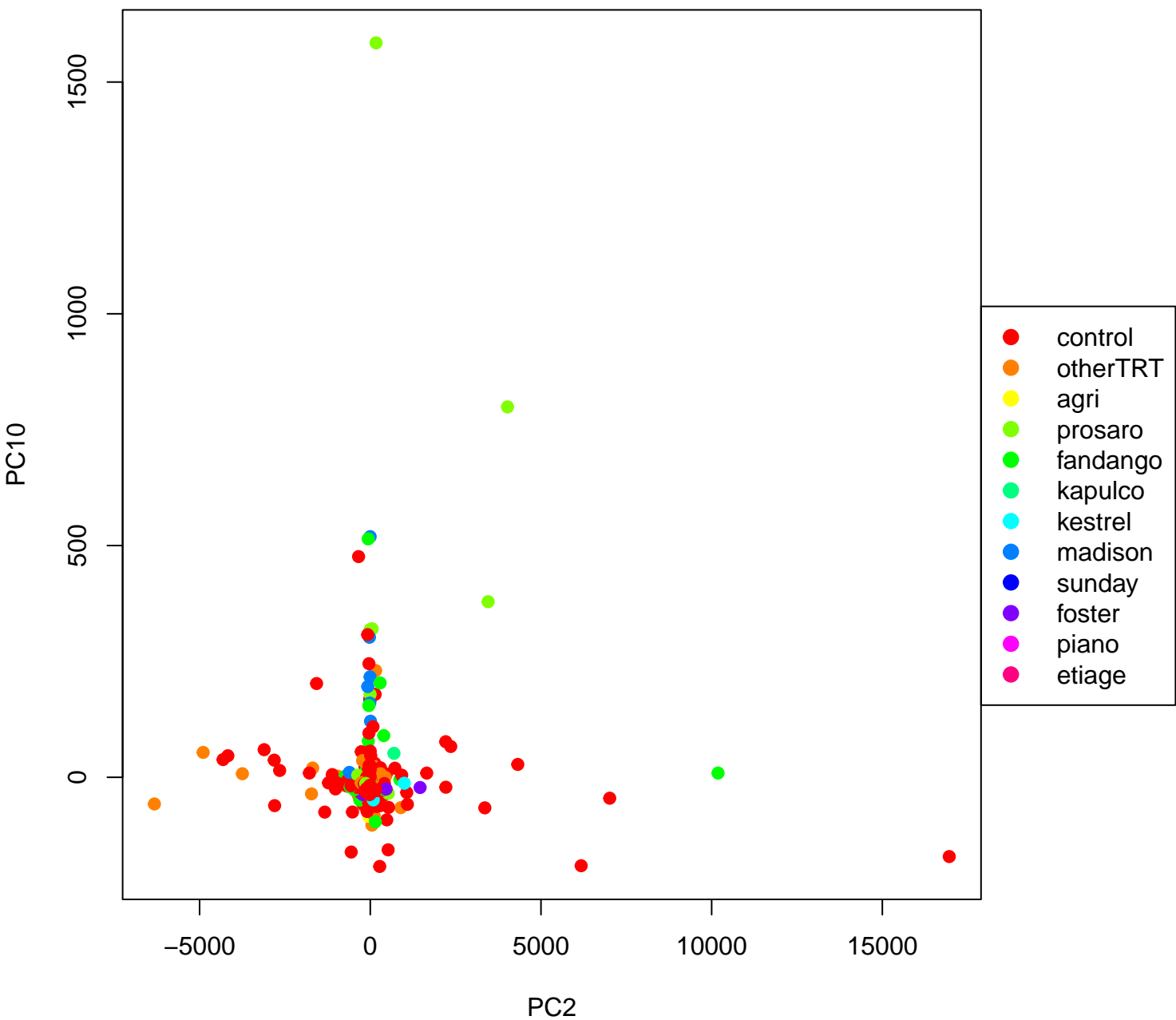

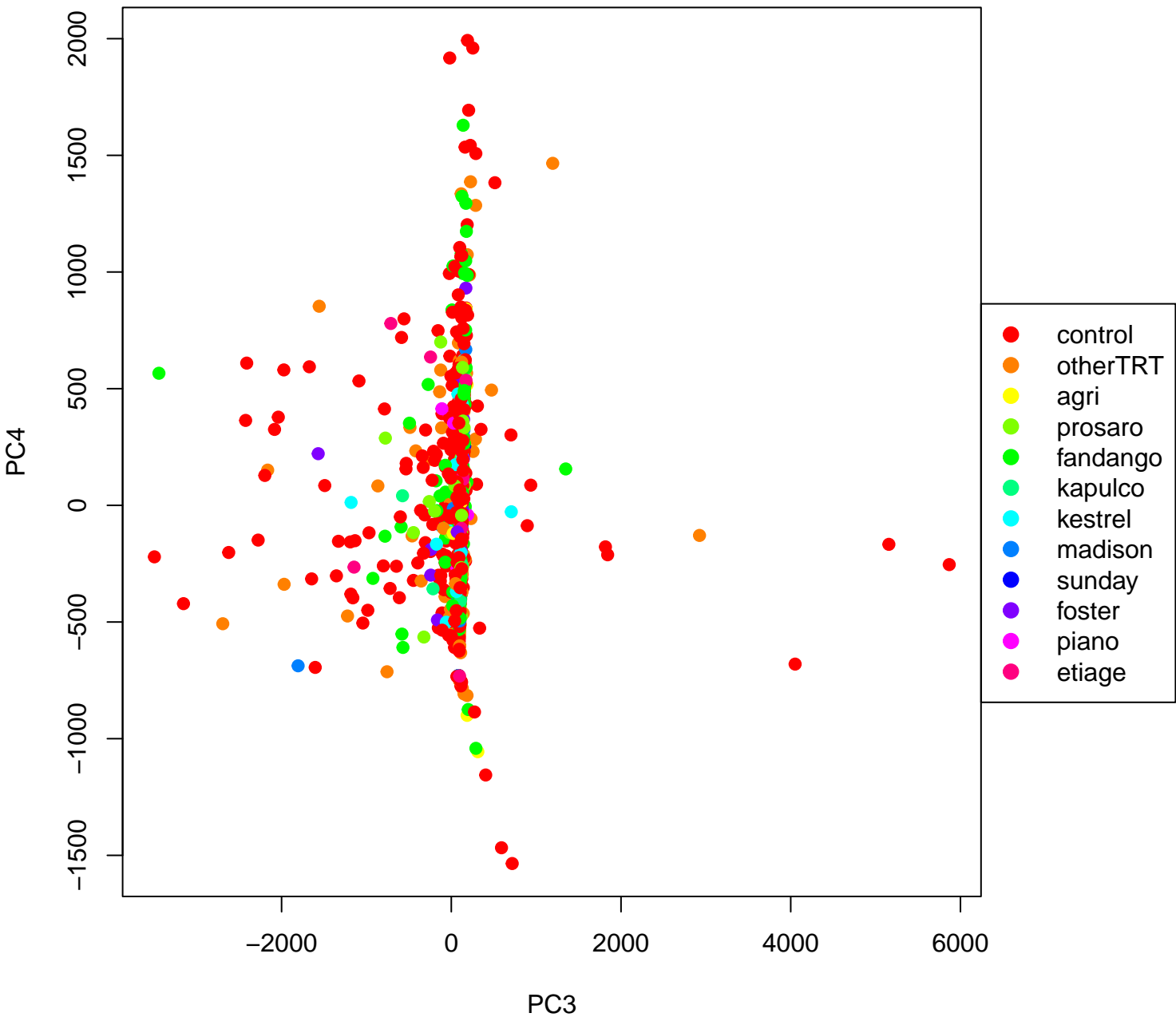

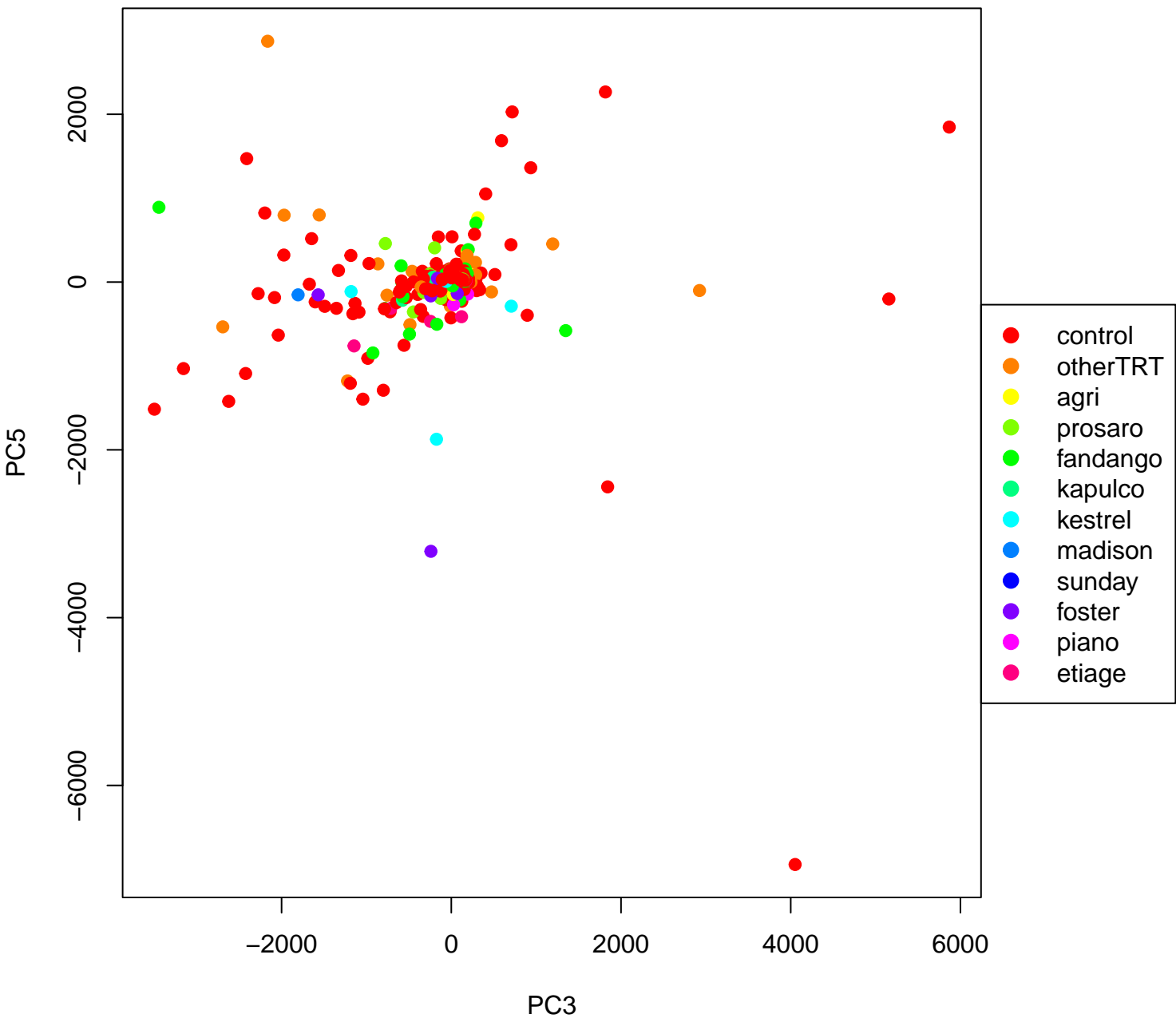

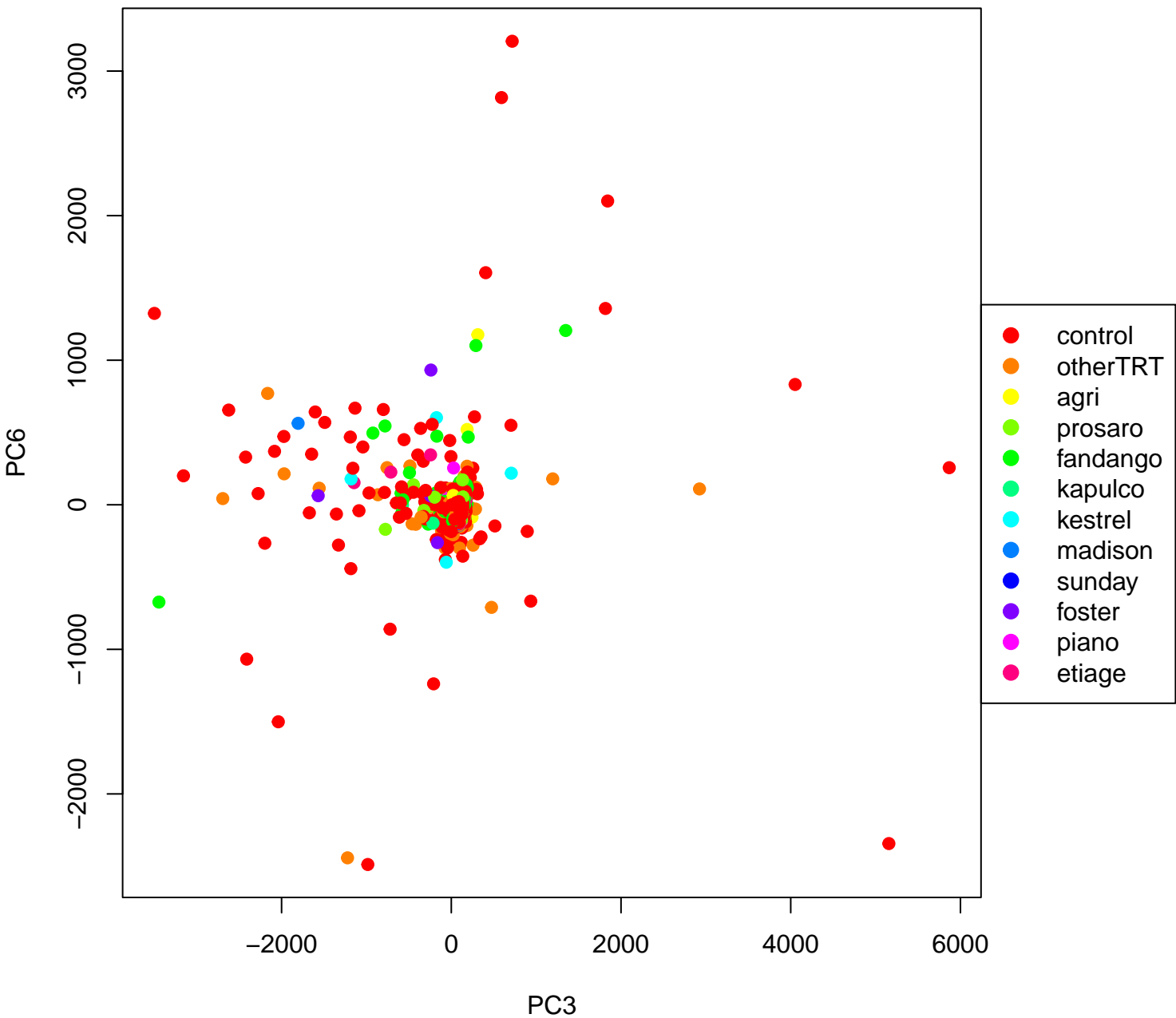

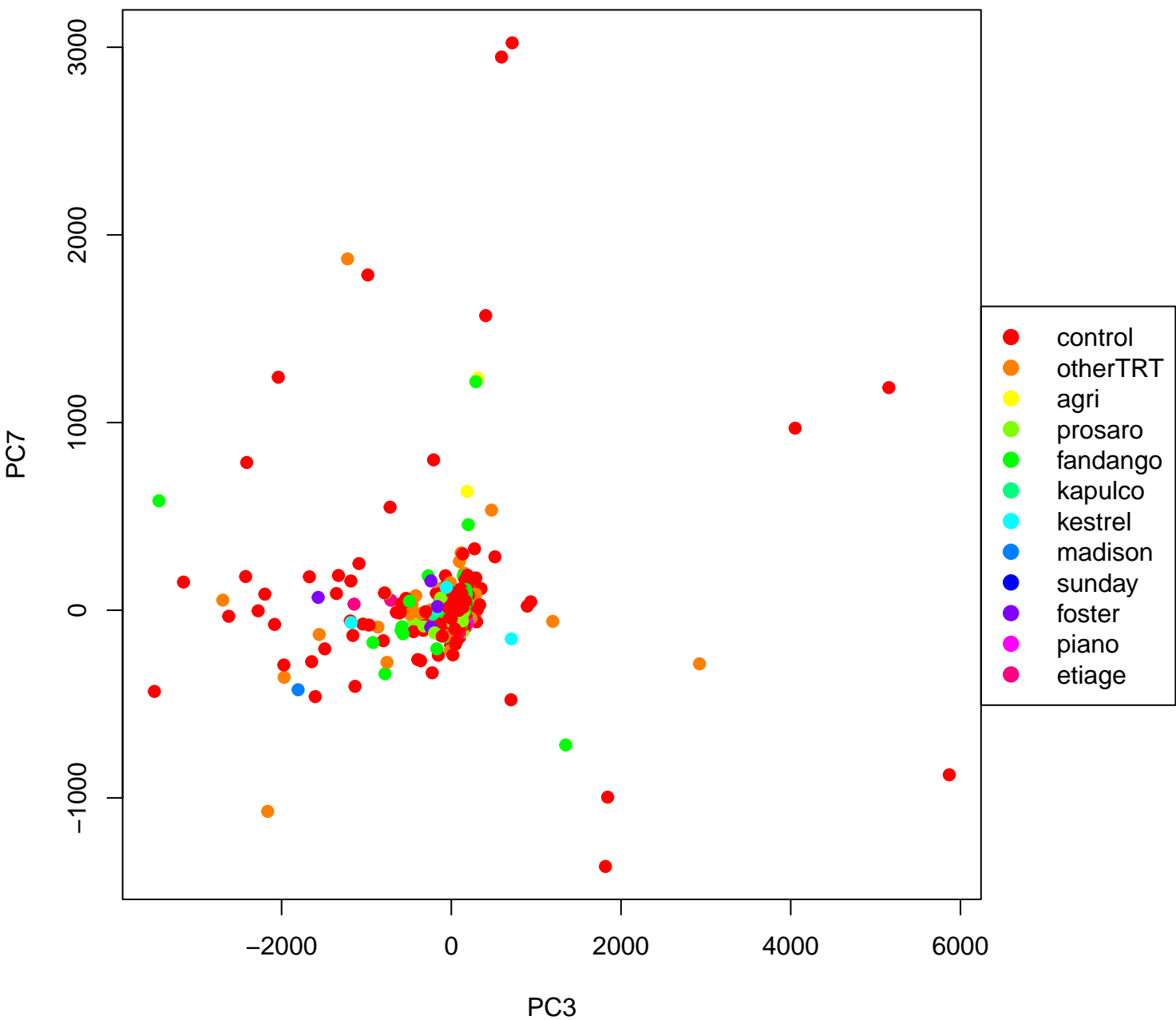

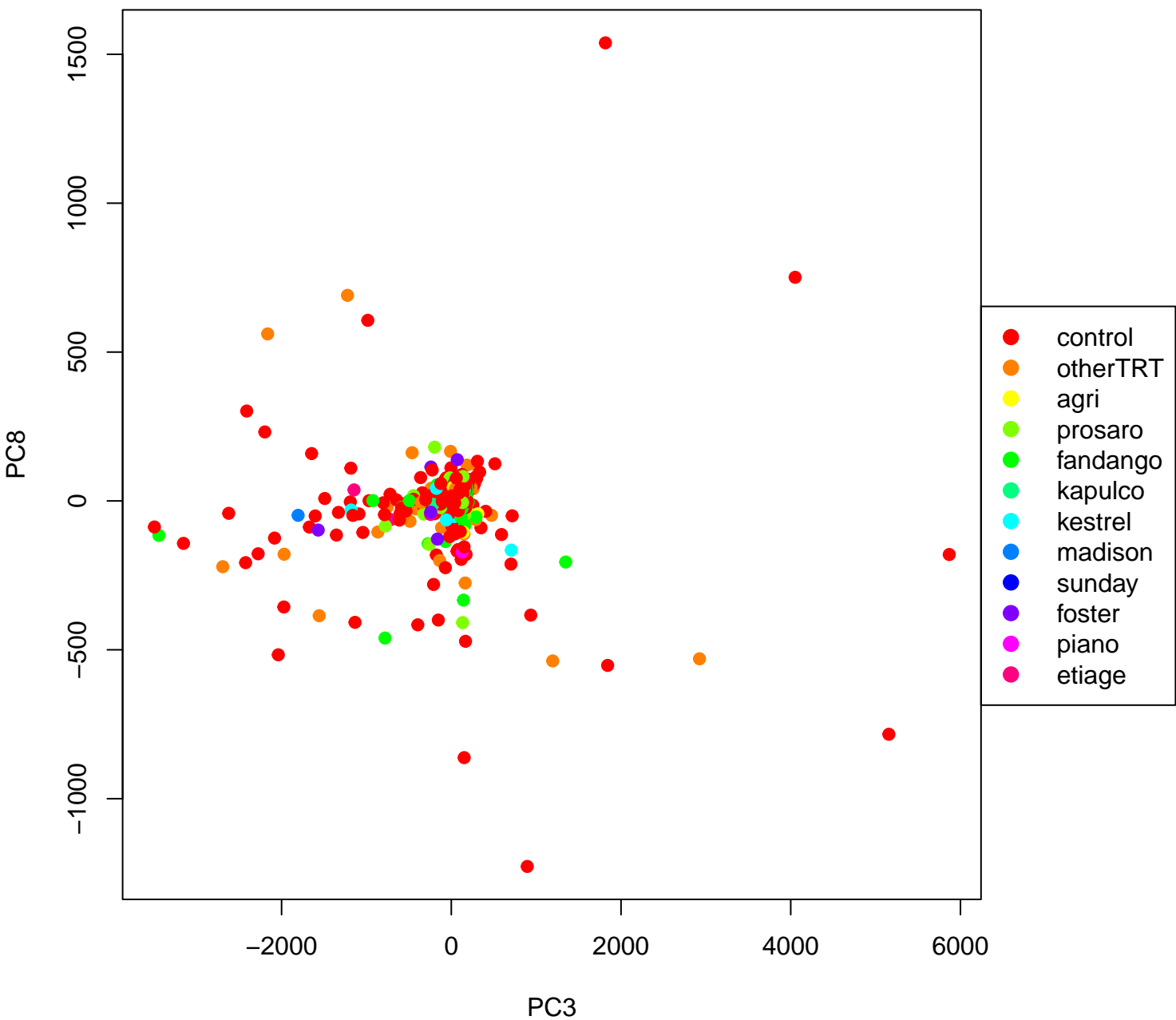

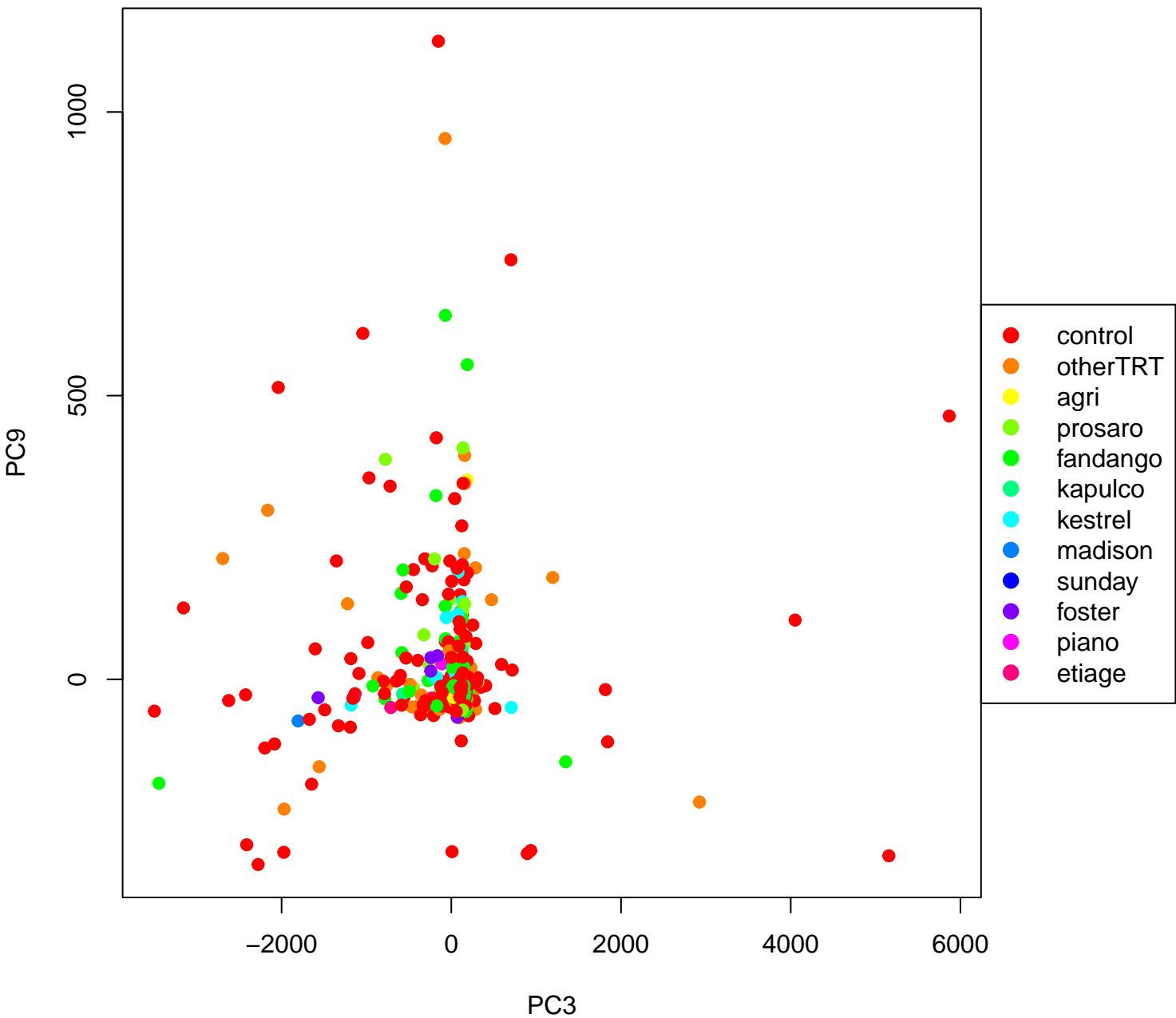

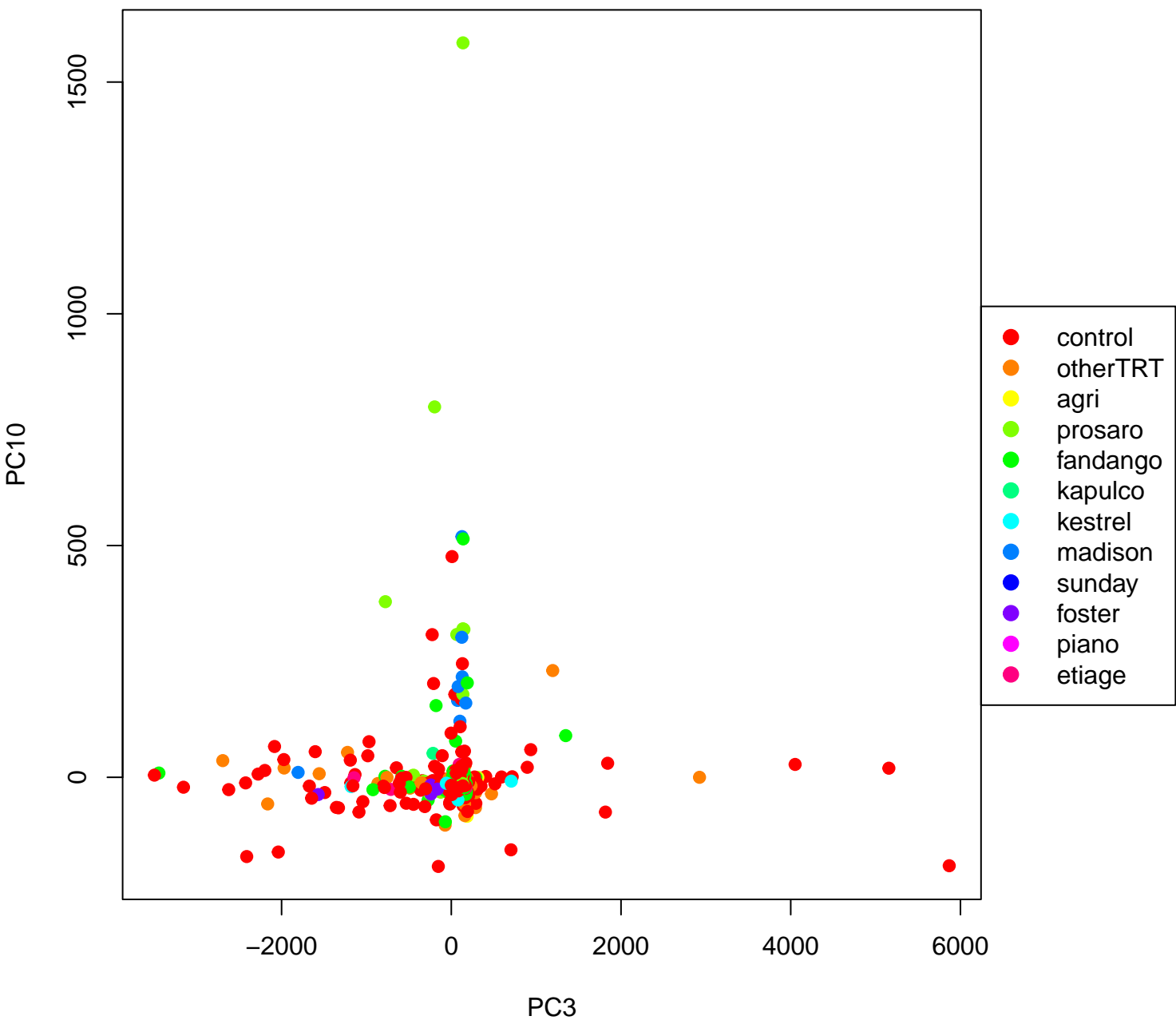

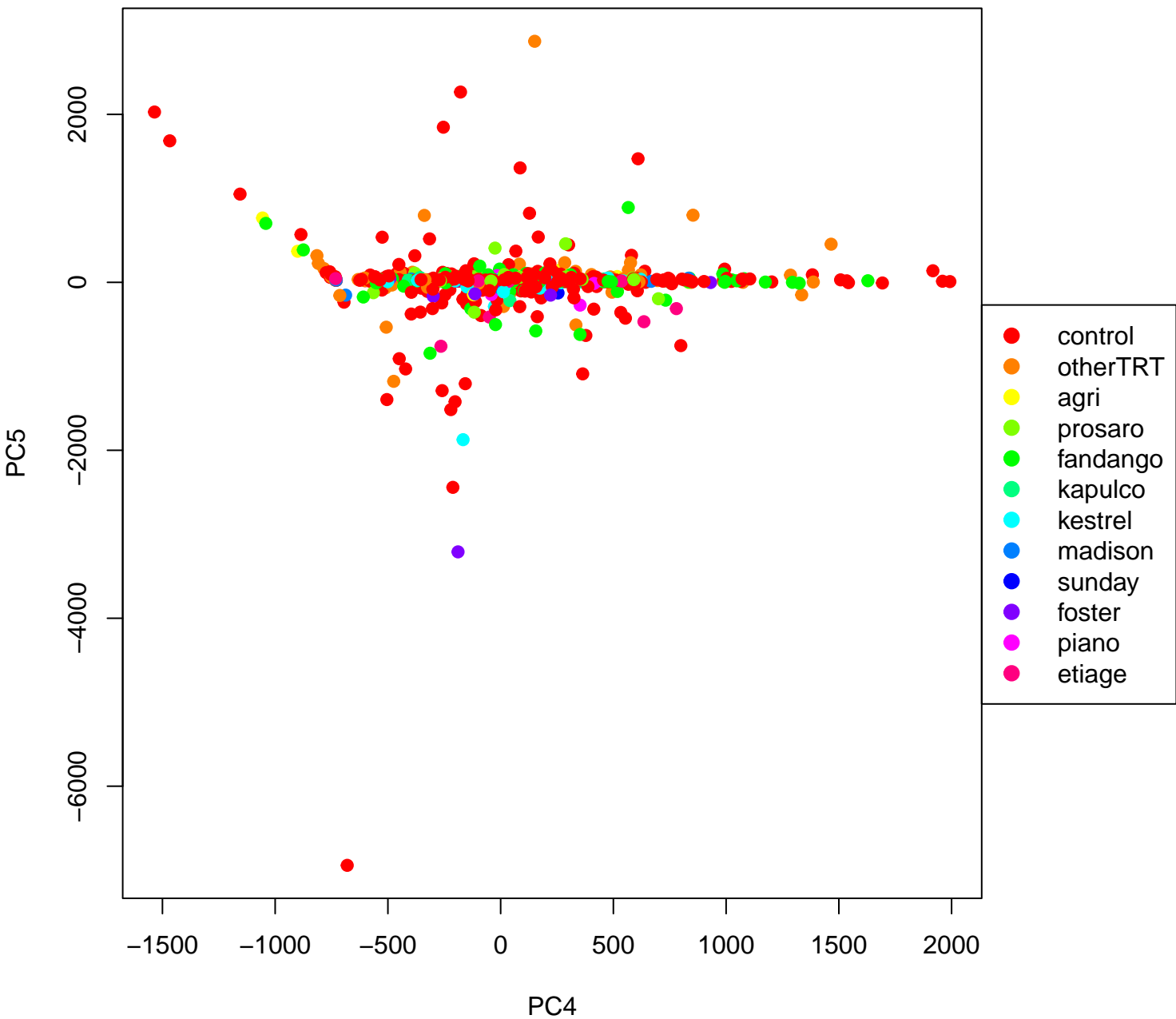

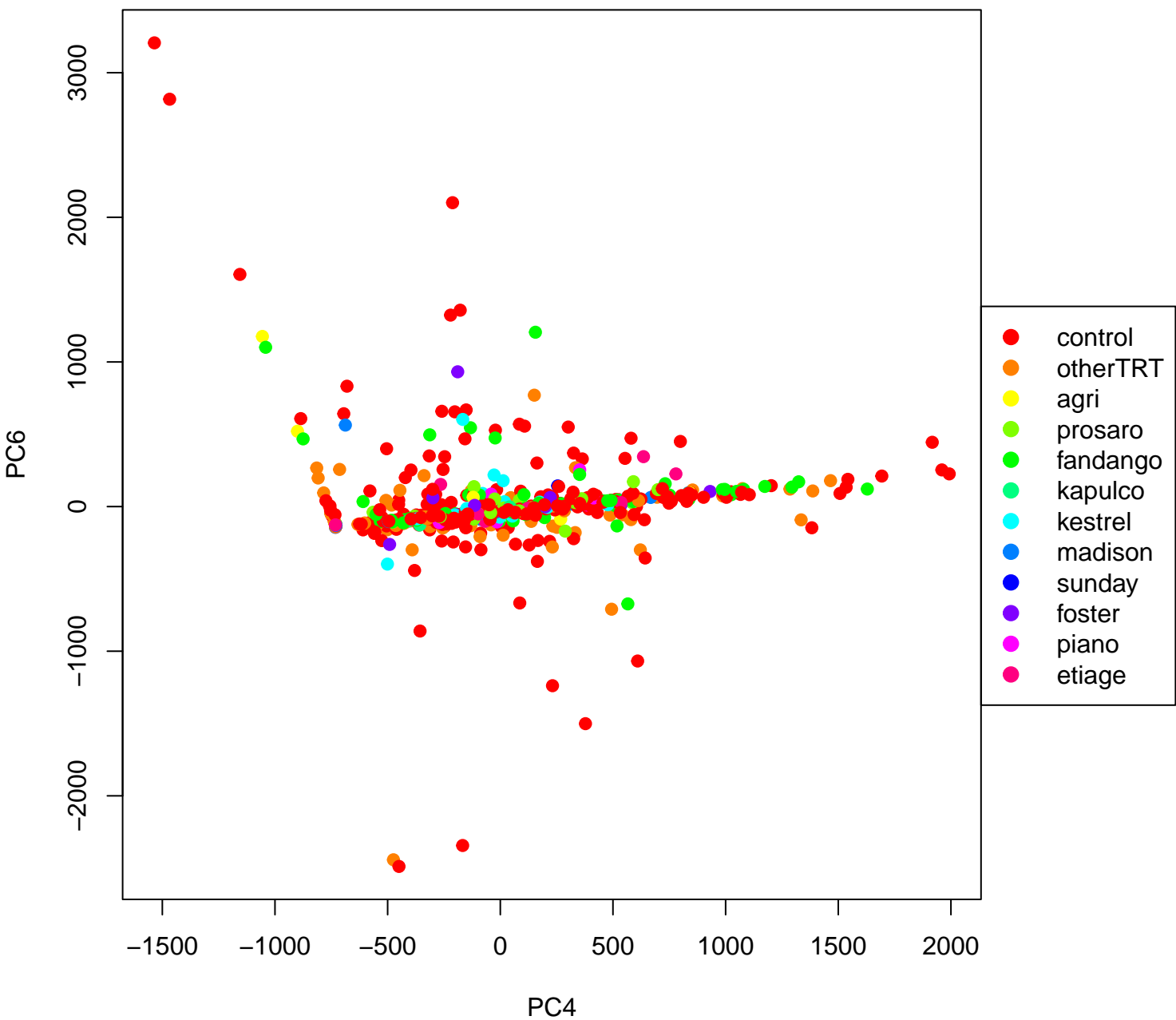

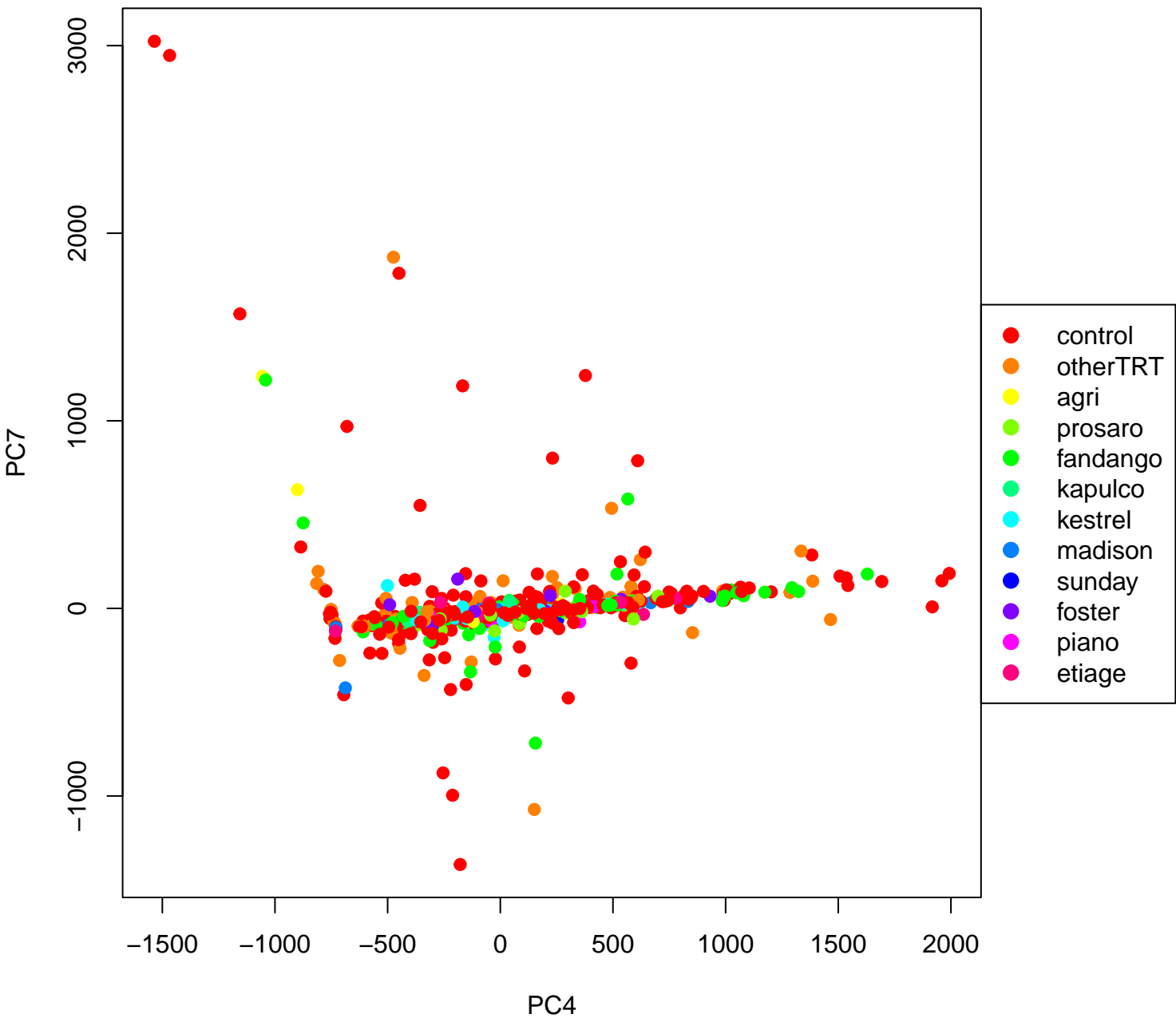

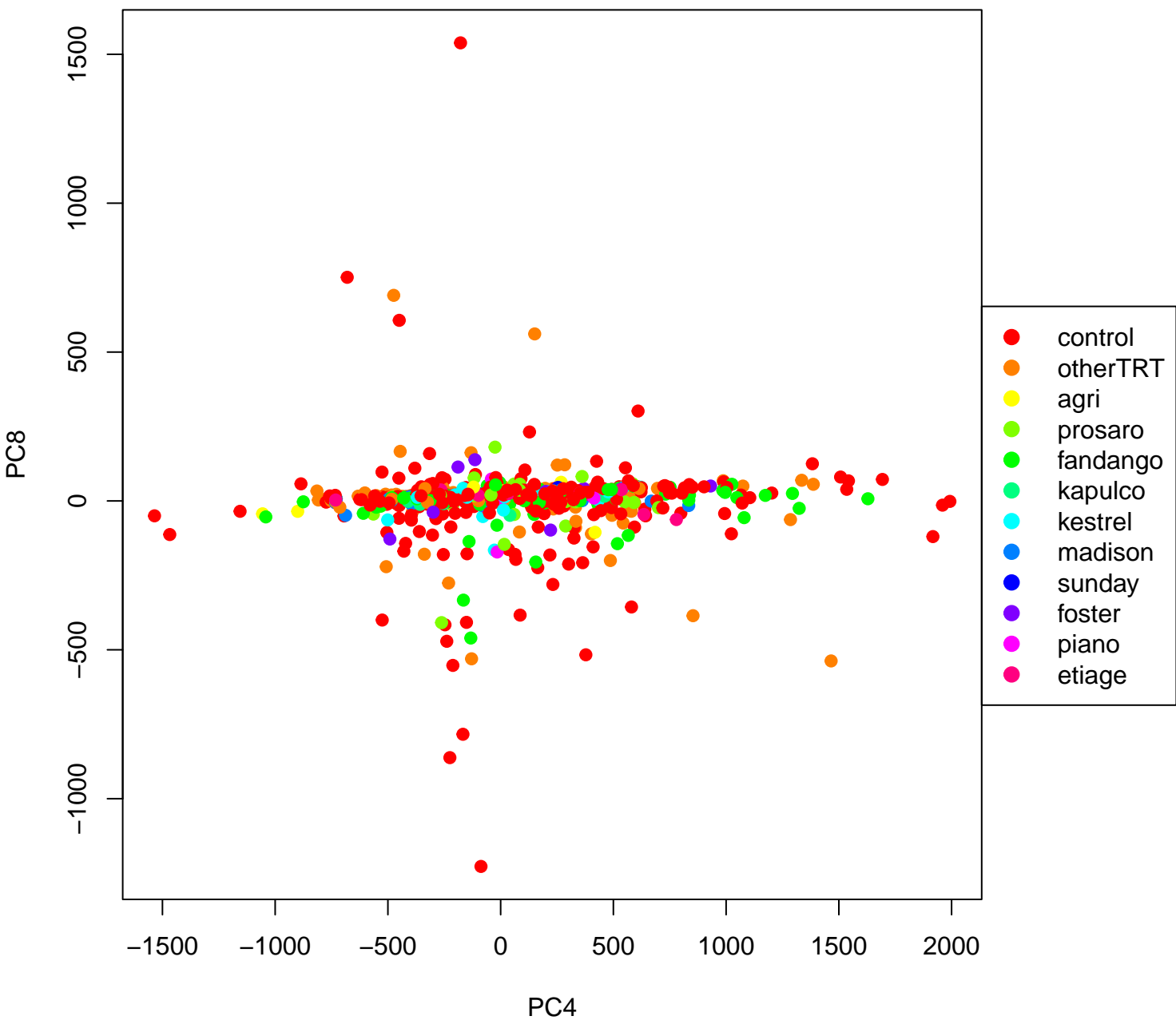

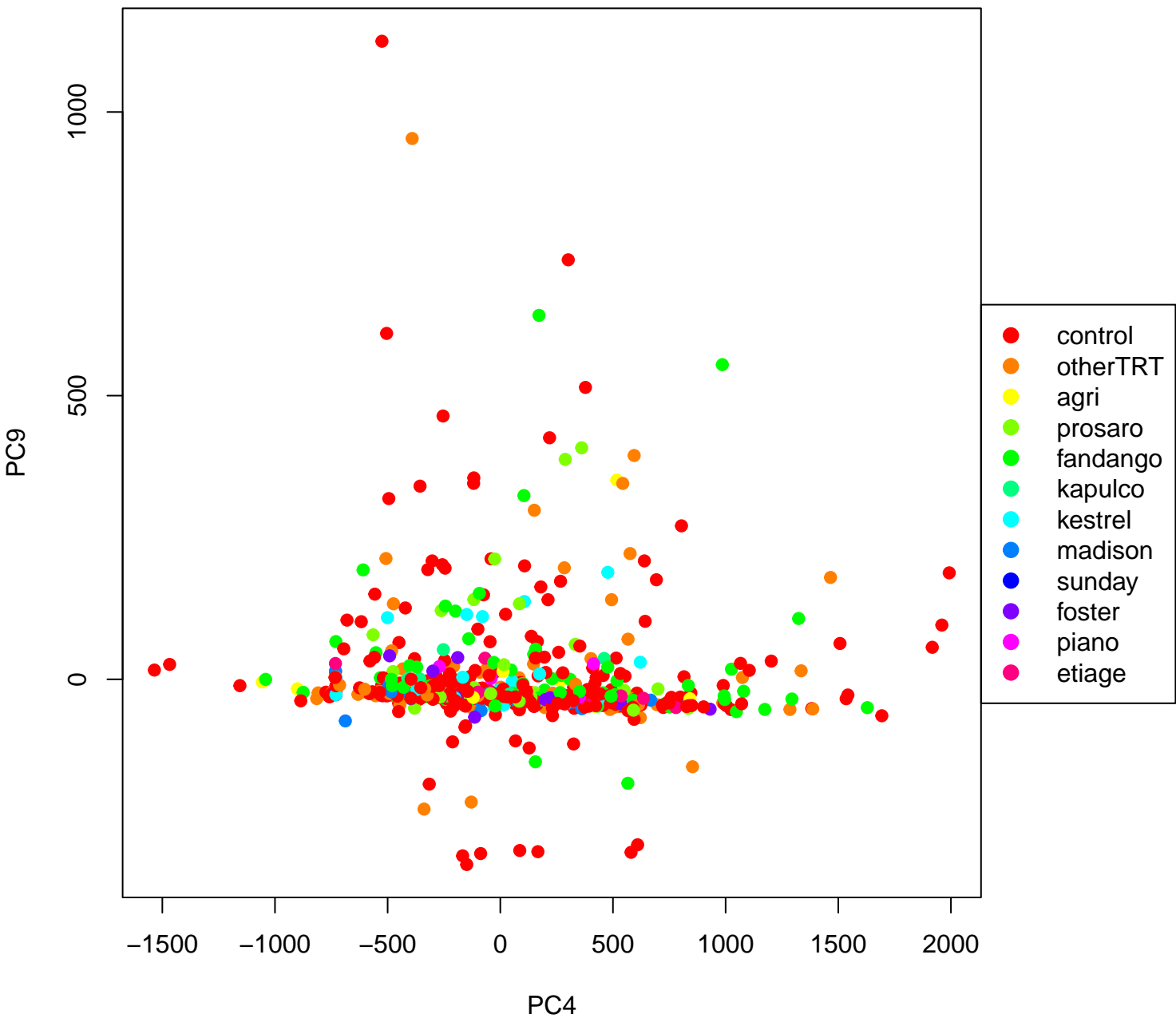

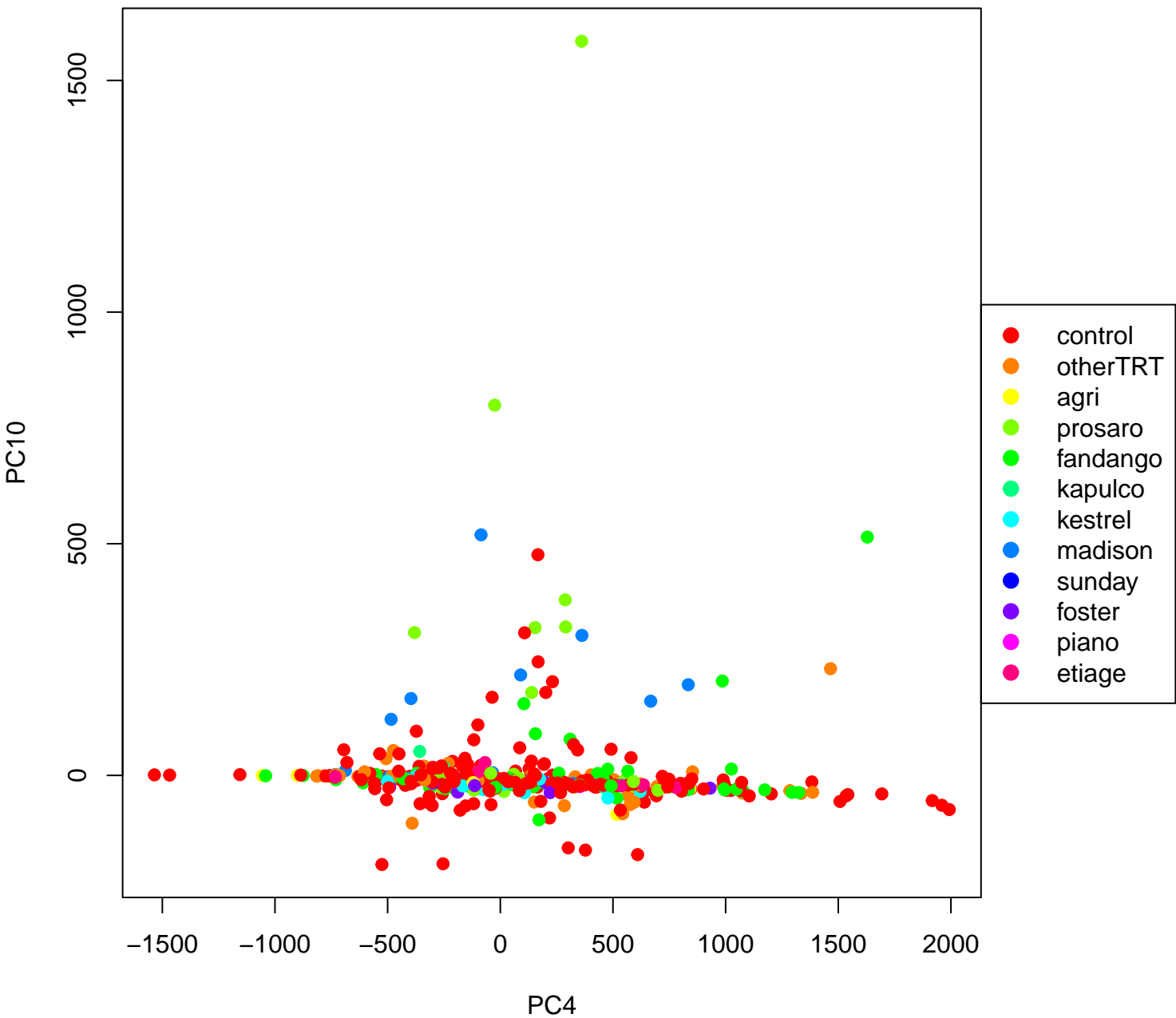

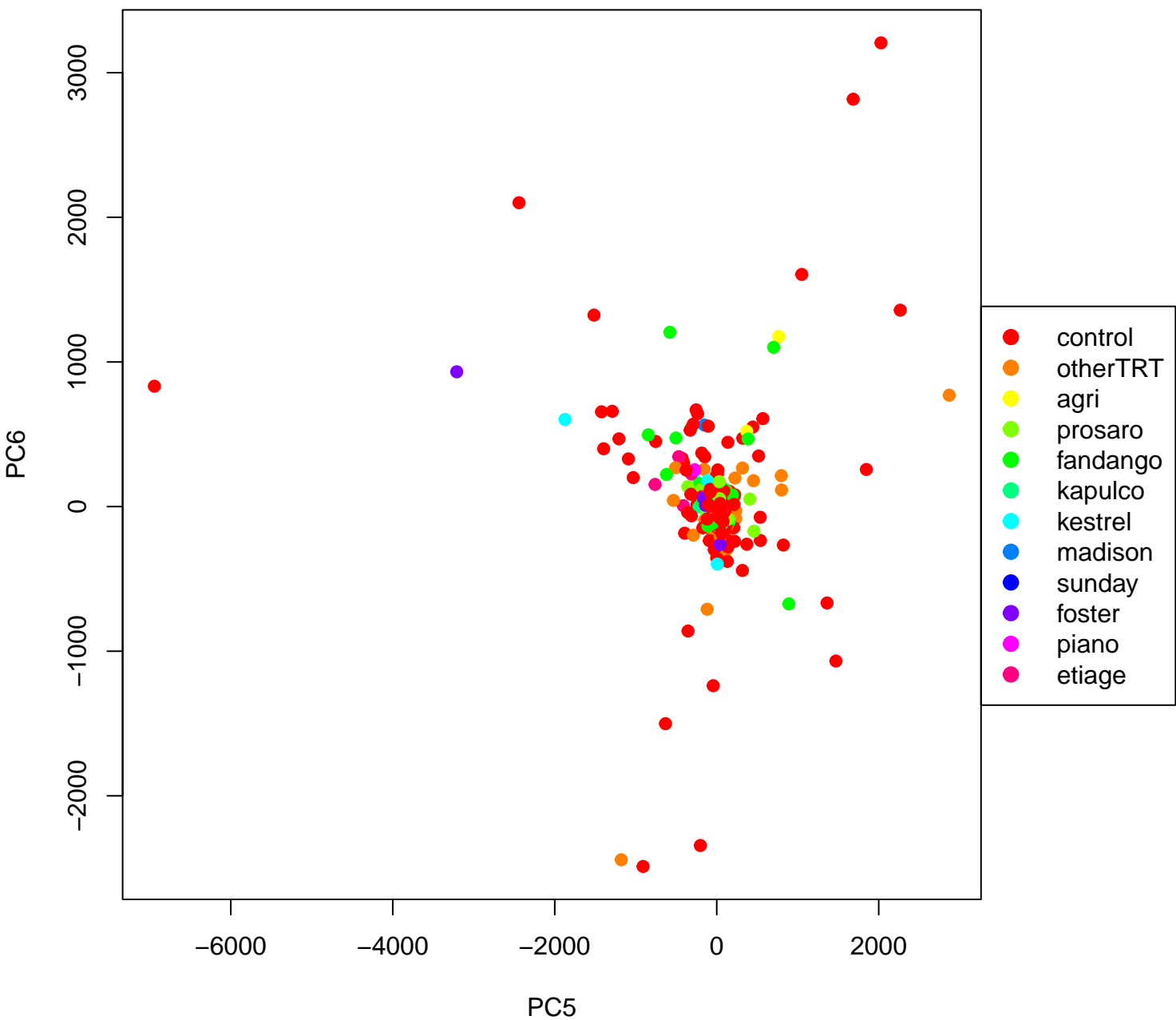

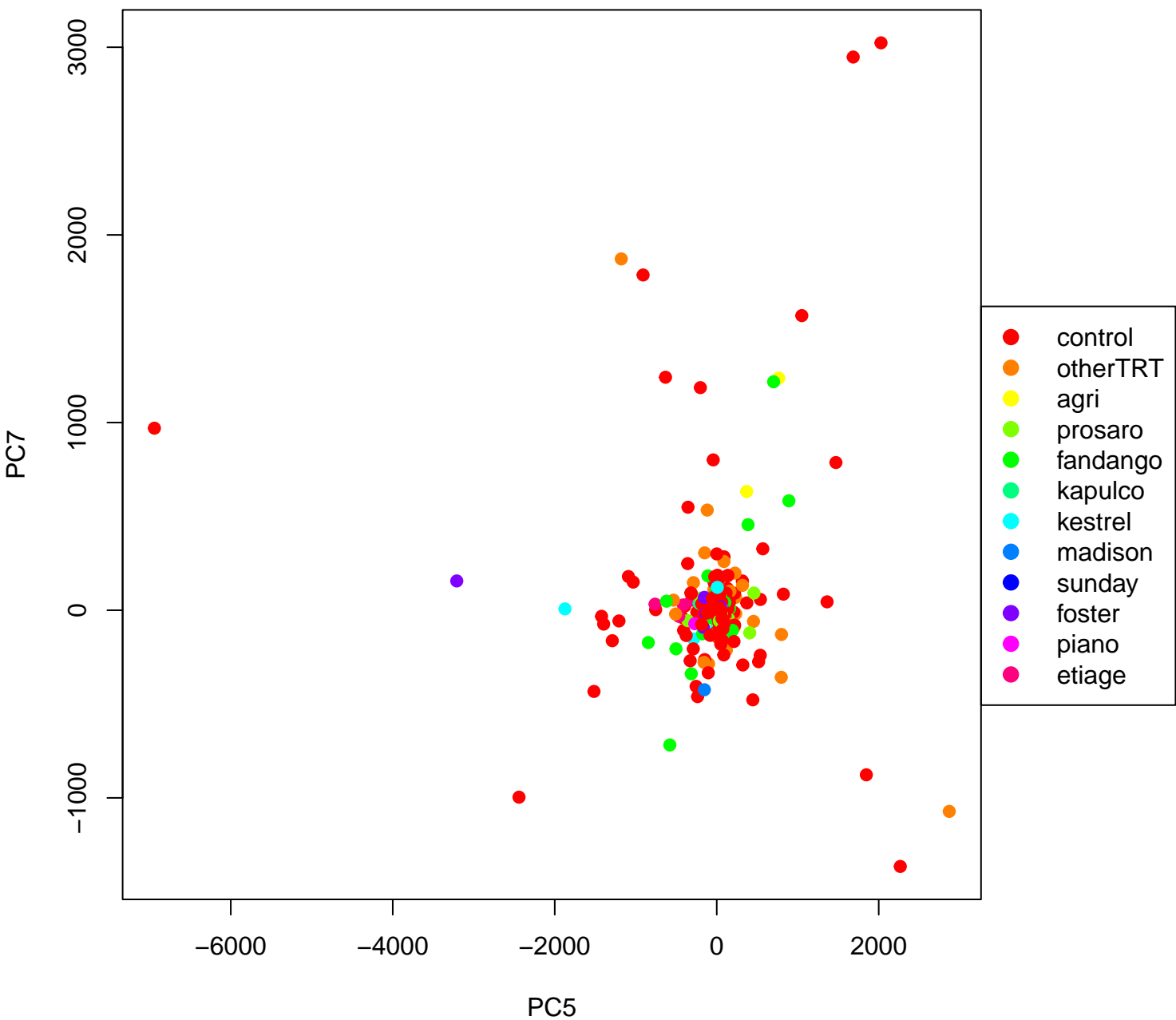

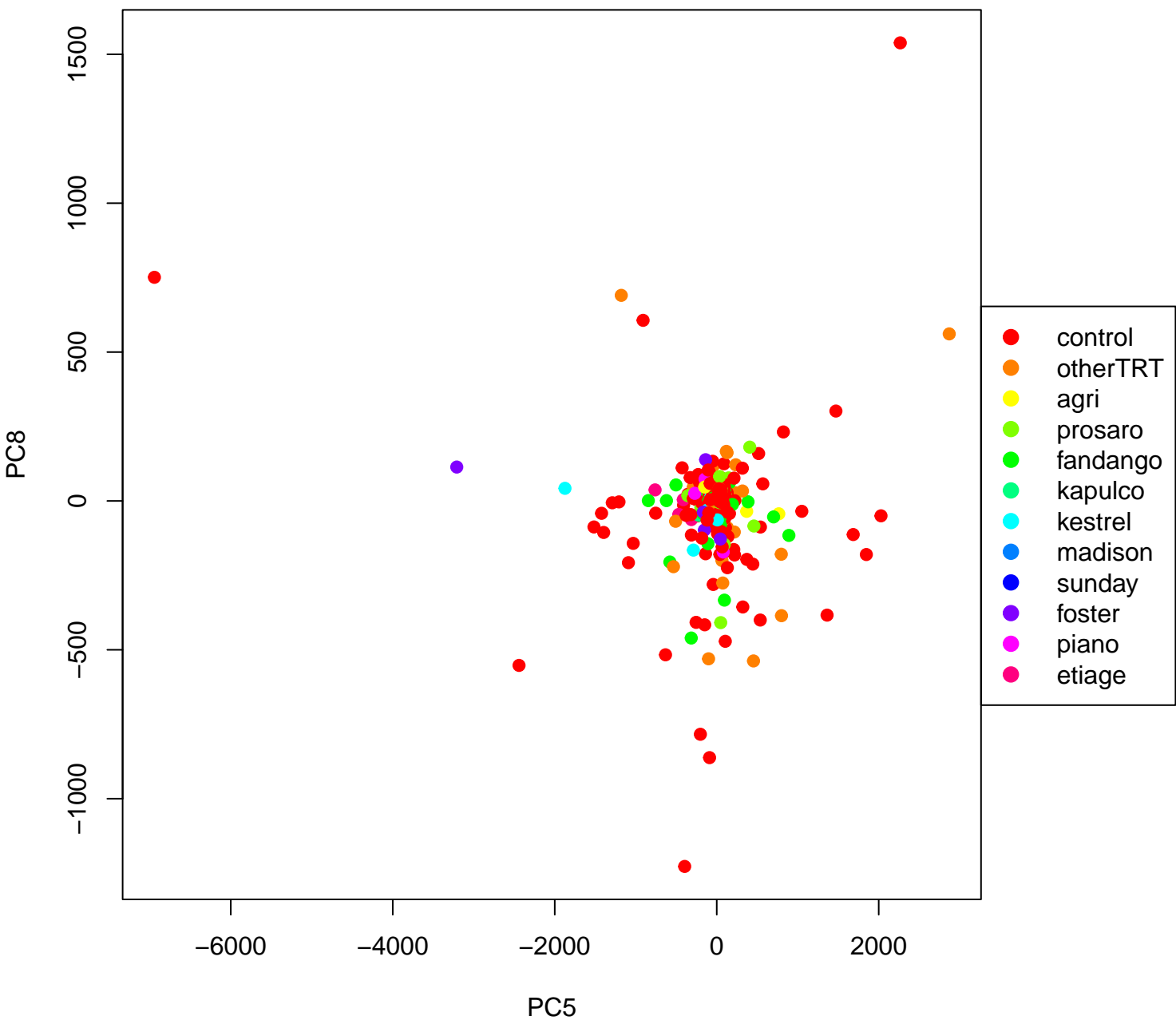

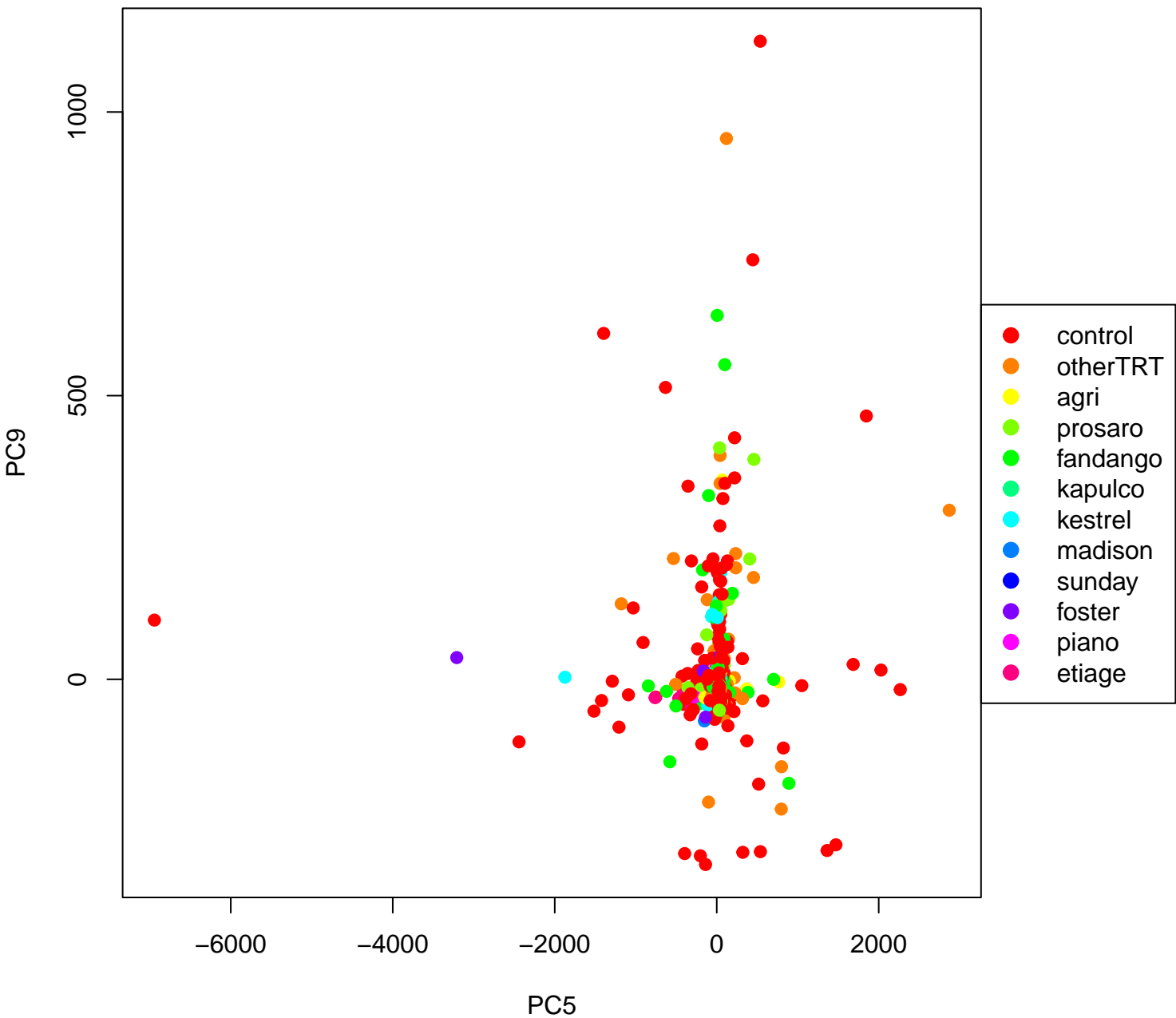

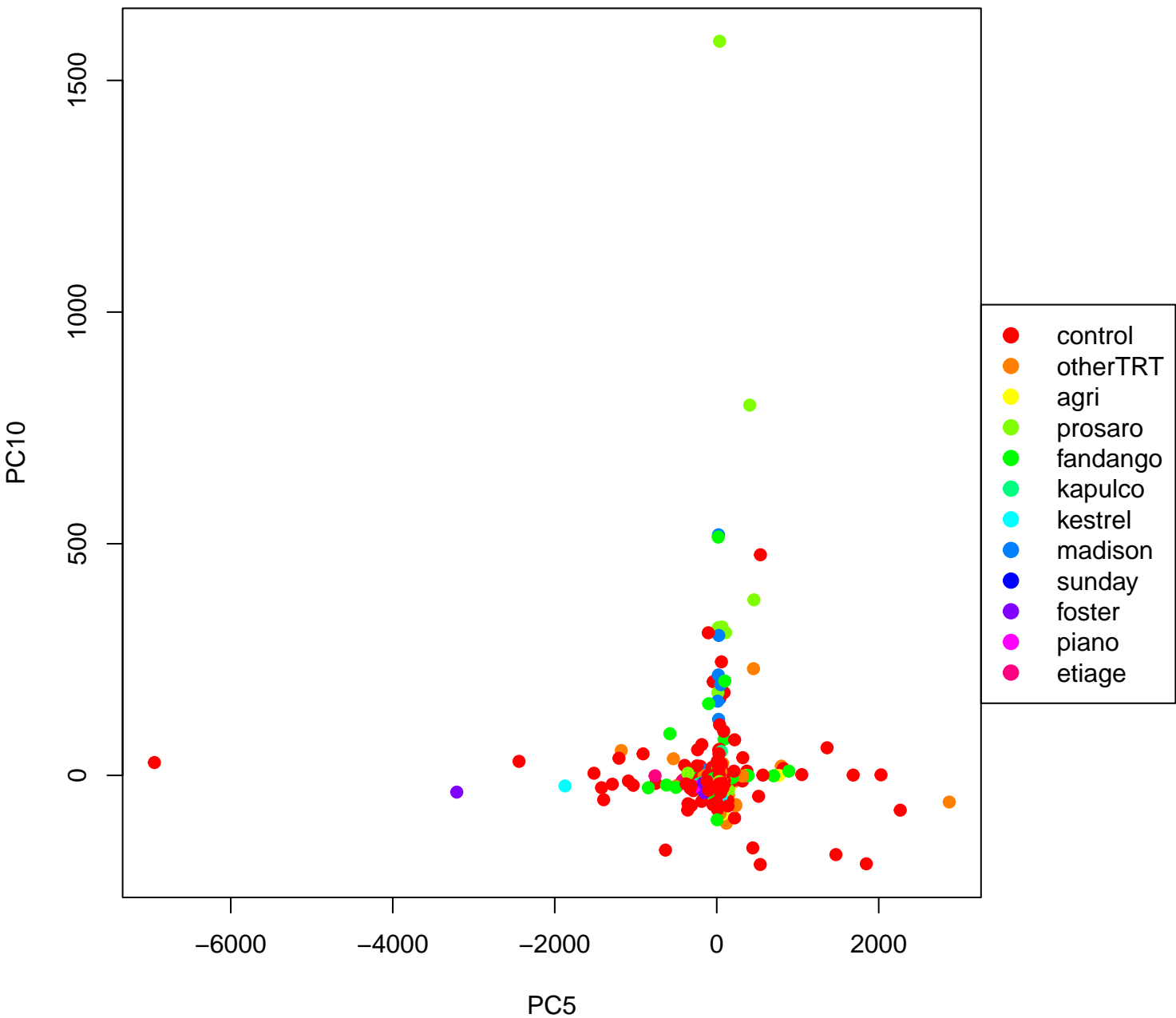

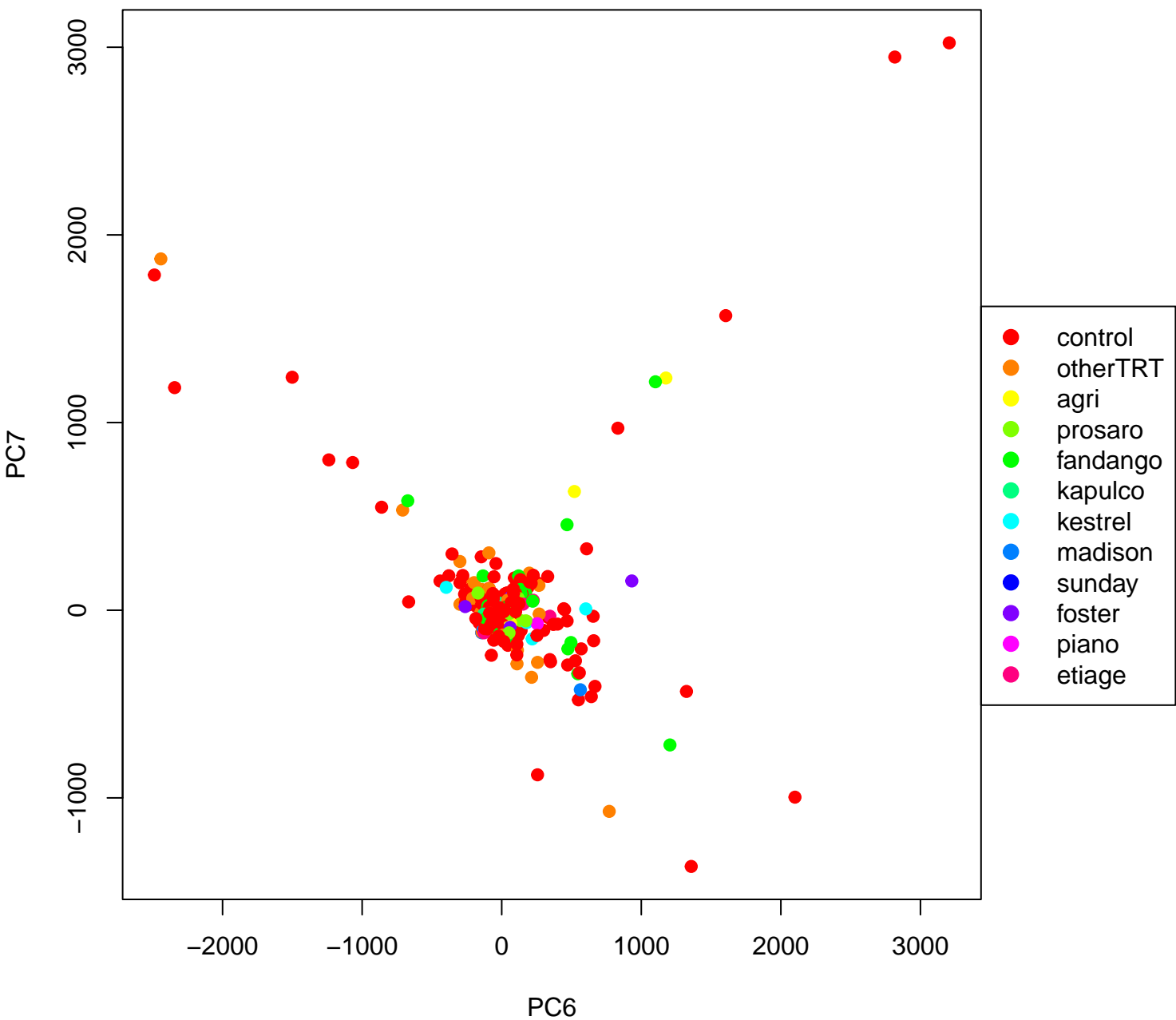

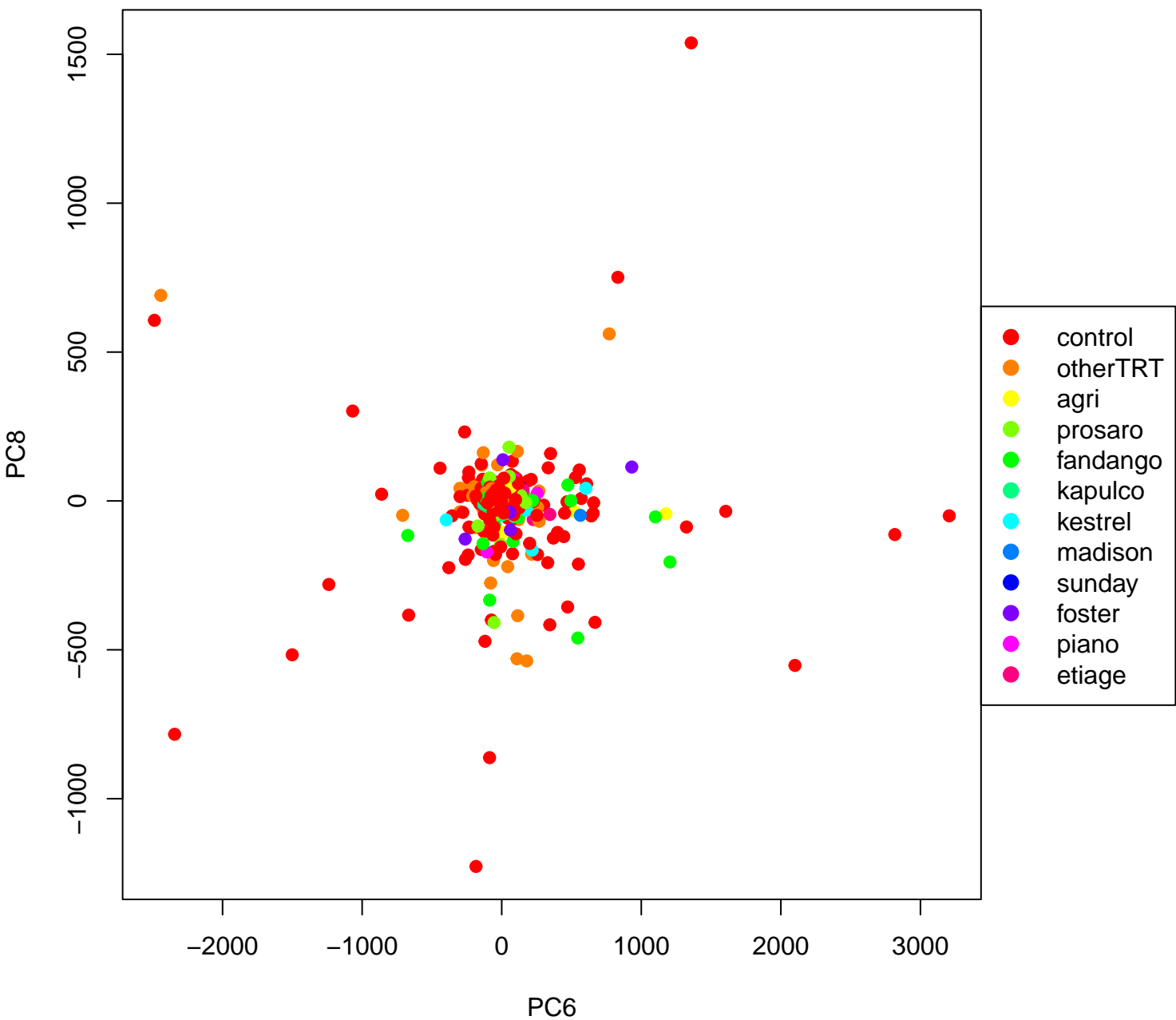

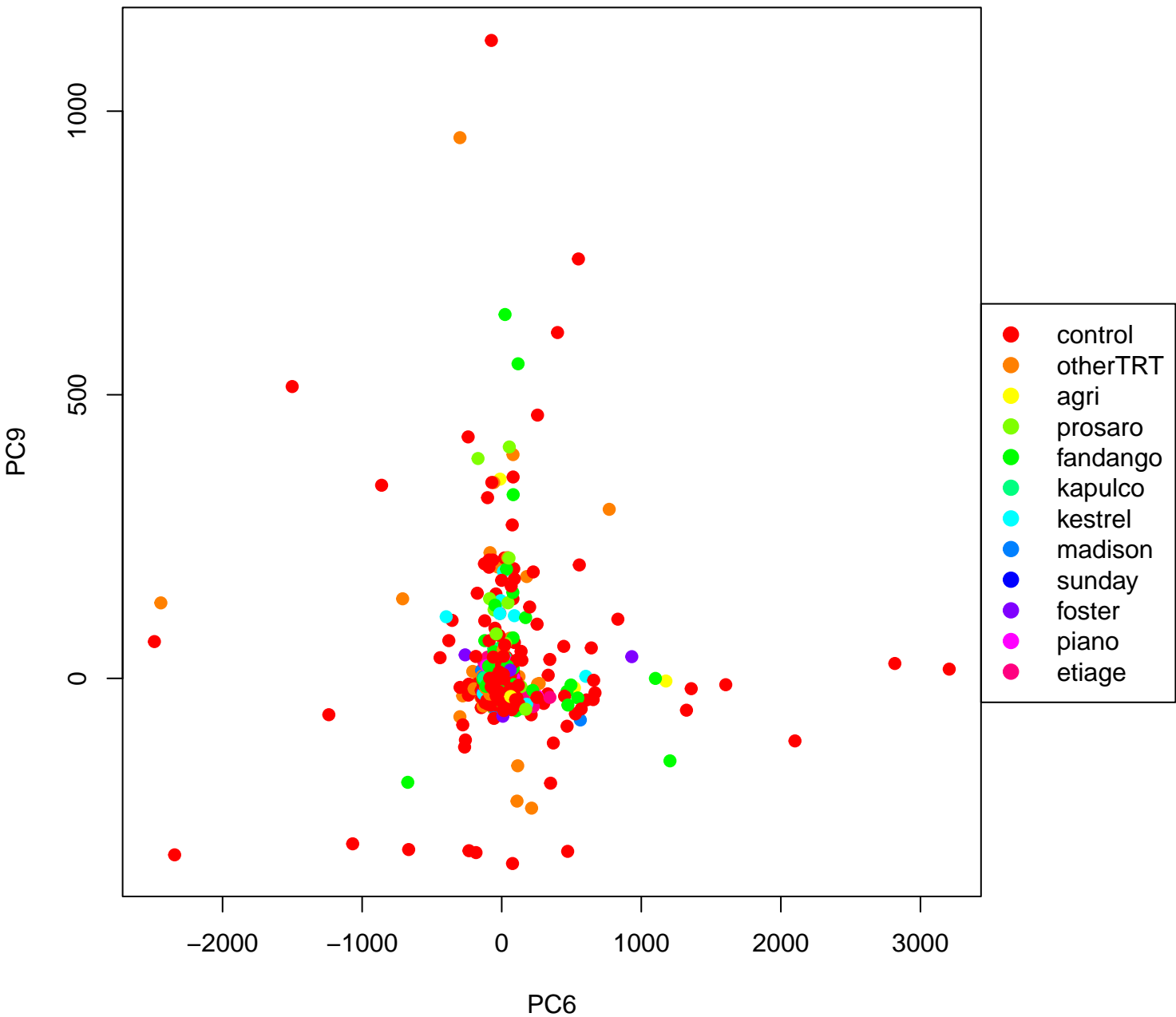

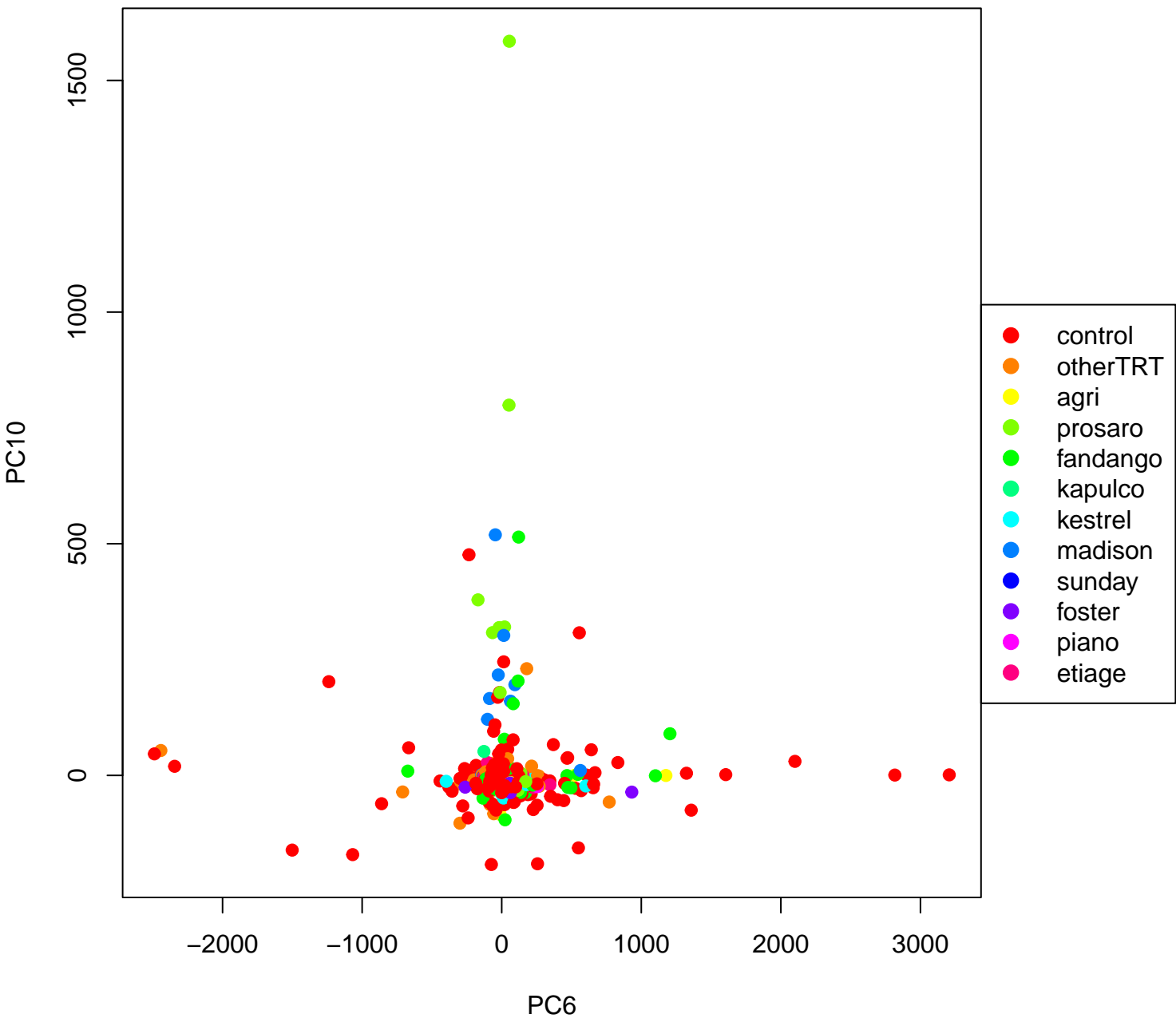

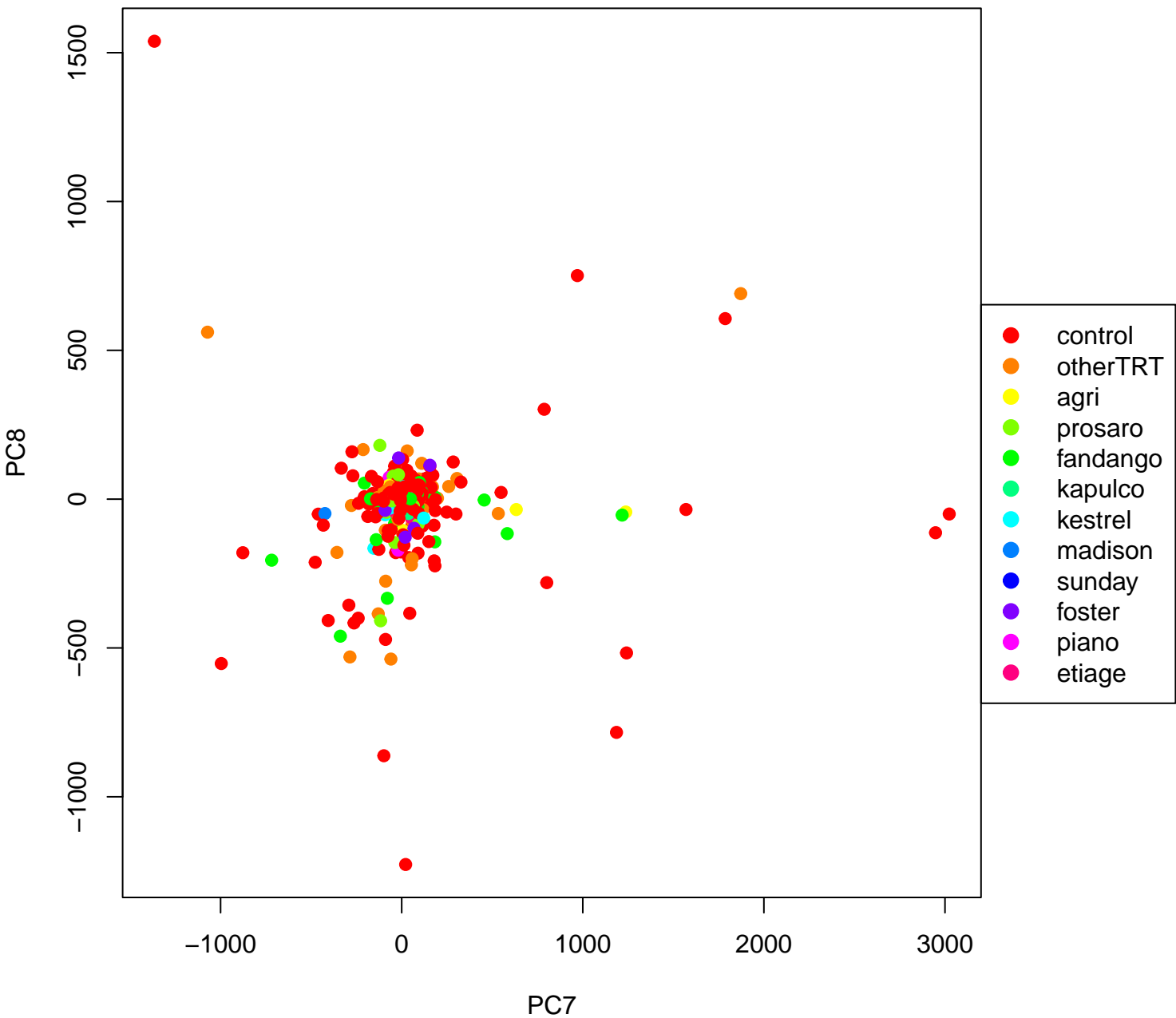

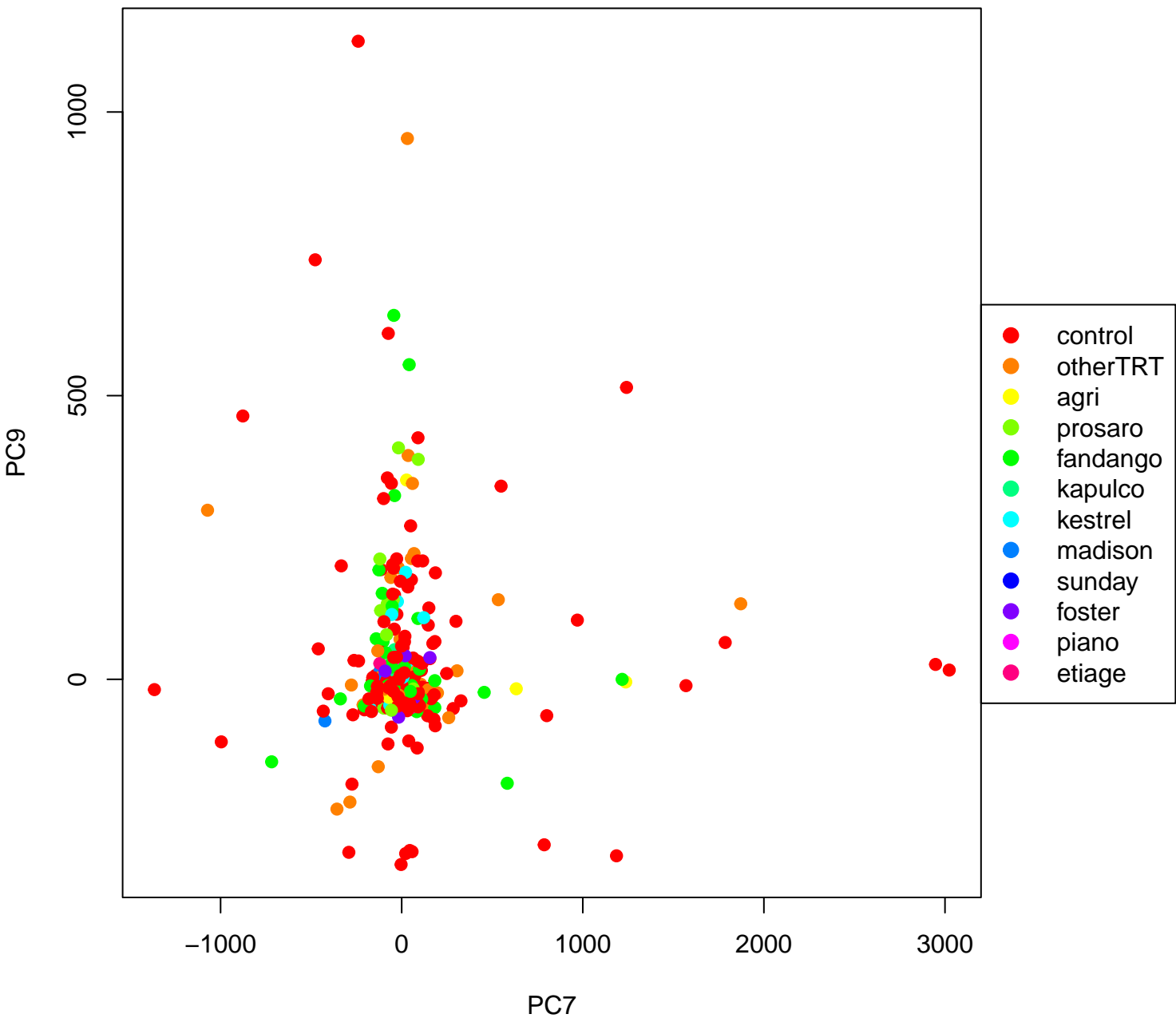

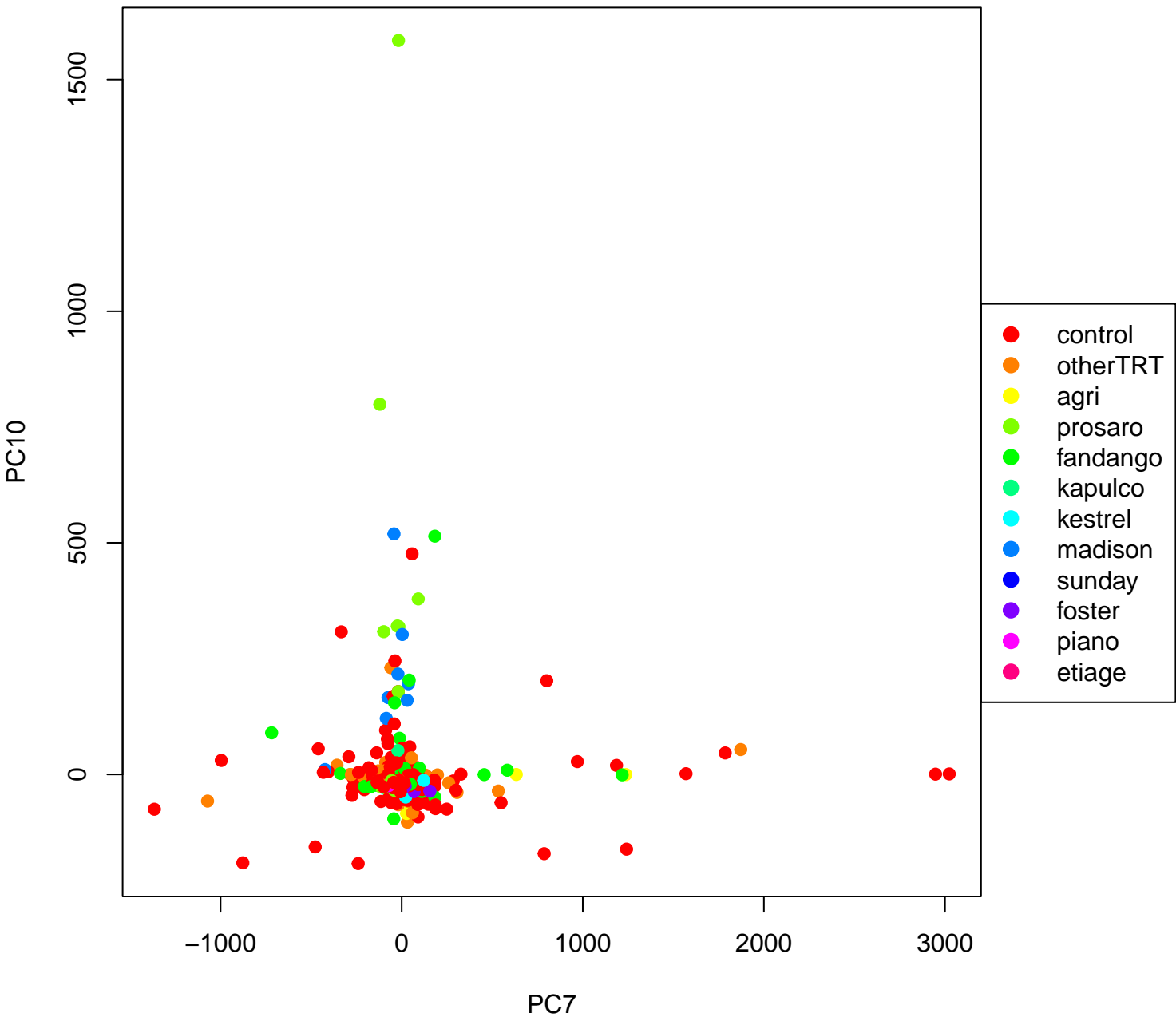

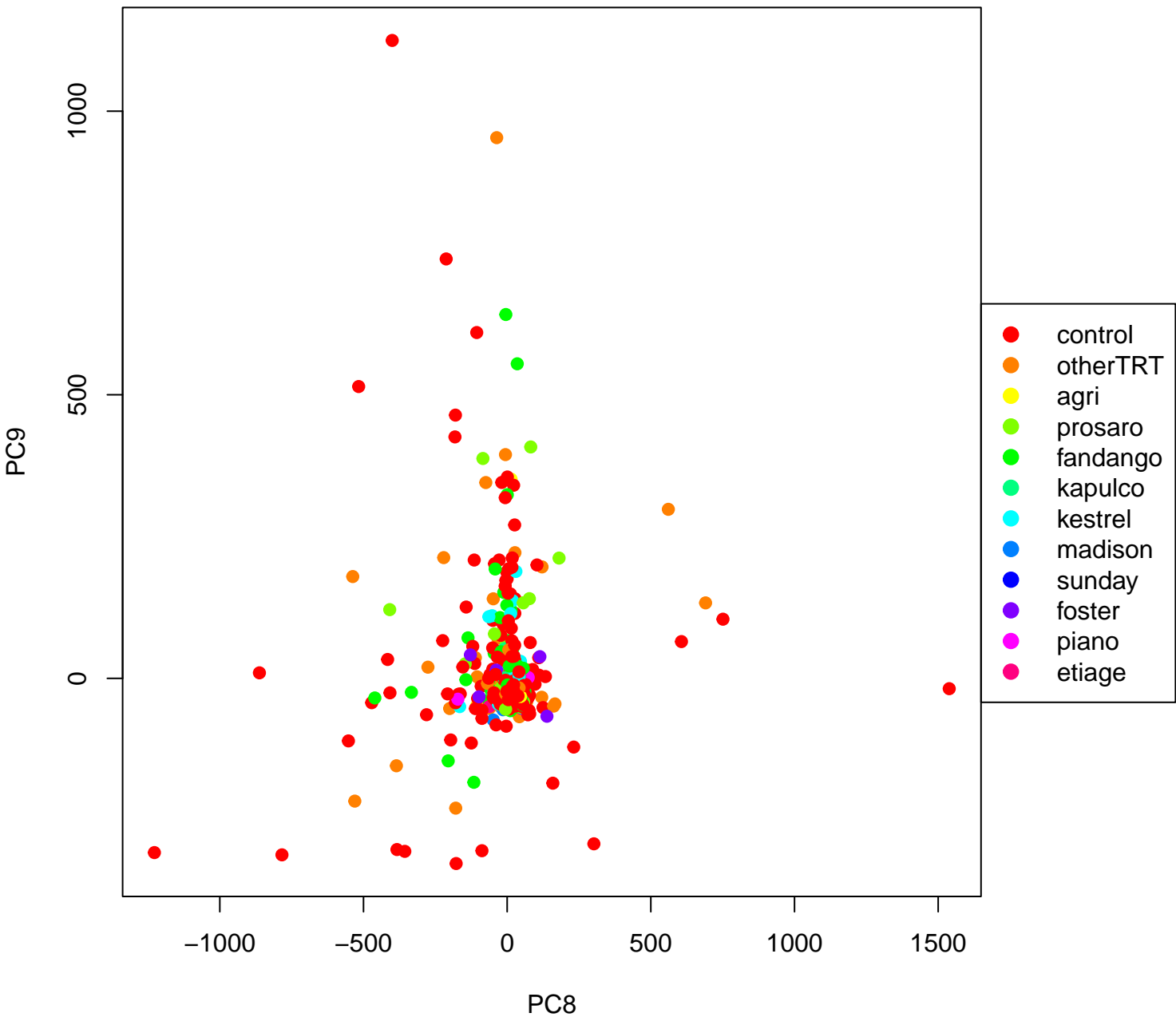

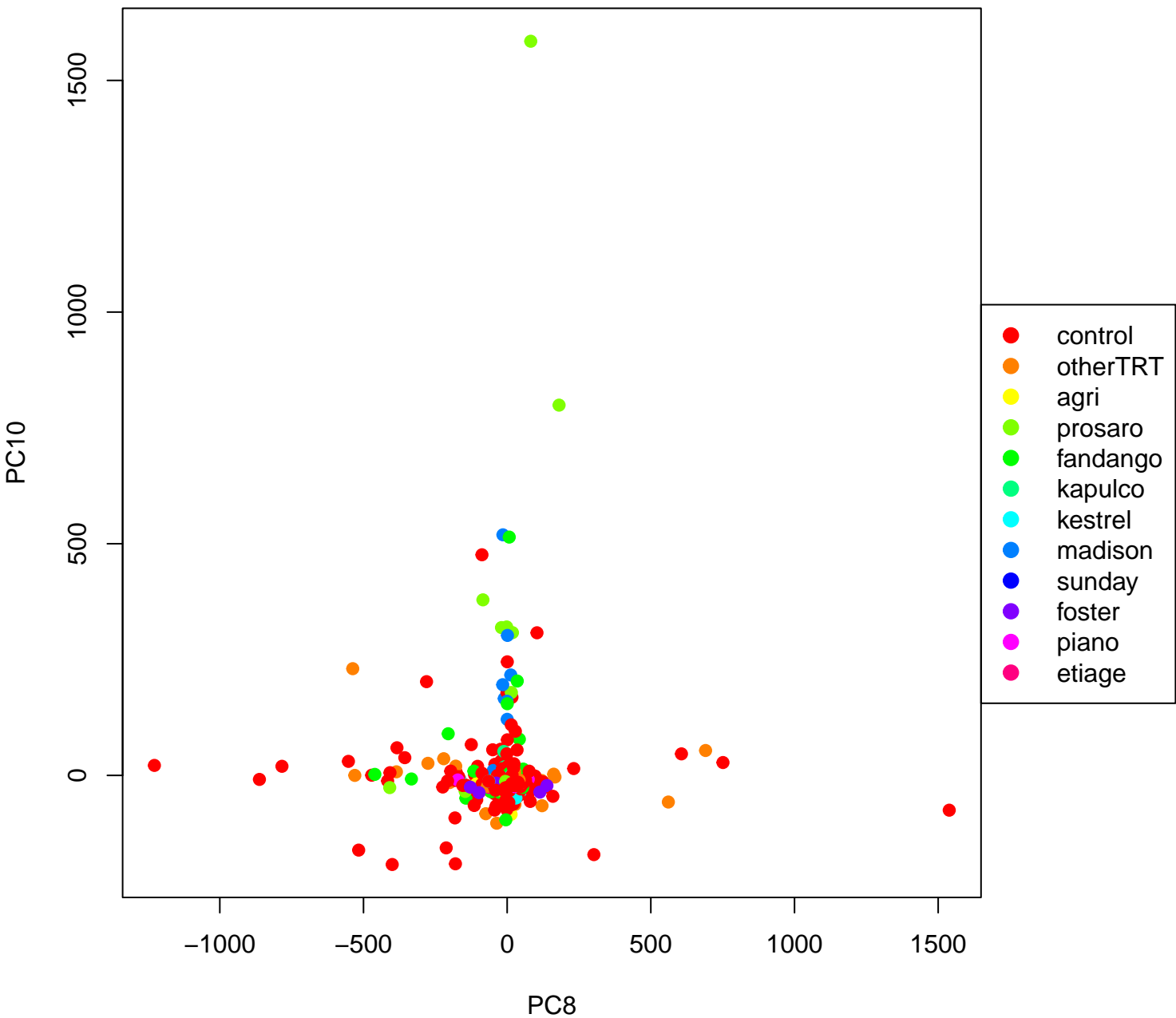

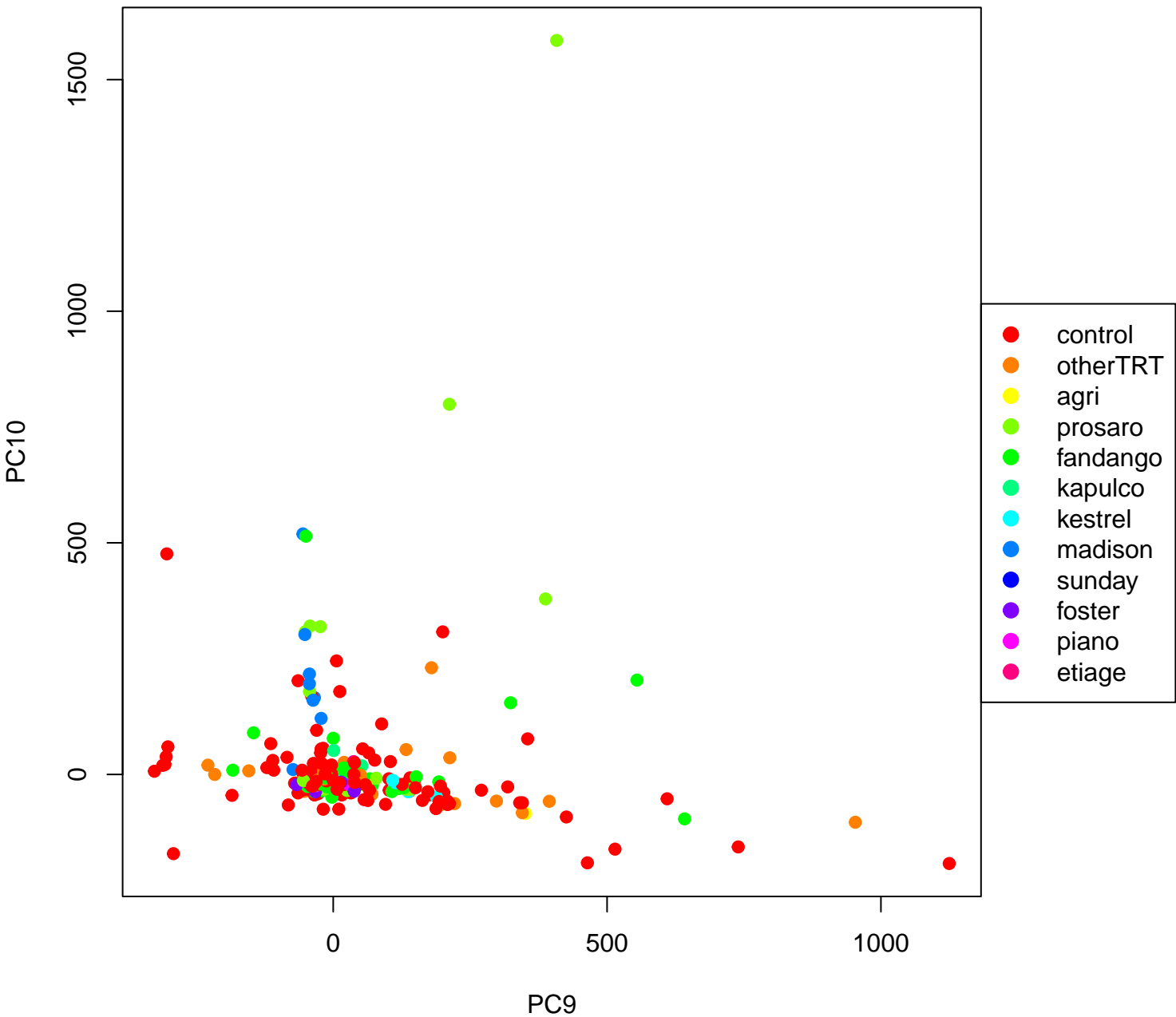

Supplement: Supplementary file 1 [file toxins-15-00443-s001.zip › Figure S1-S45_PCA and hierarchical clustering for the investigation of potential biases of individual fungicides.pdf]
